# Integrated Transcriptomic and Proteomic Analyses Reveal CsrA-Mediated Regulation of Virulence and Metabolism in *Vibrio alginolyticus*

**DOI:** 10.3390/microorganisms13071516

**Published:** 2025-06-28

**Authors:** Bing Liu, Huizhen Chen, Kai Sheng, Jianxiang Fang, Ying Zhang, Chang Chen

**Affiliations:** 1 CAS Key Laboratory of Tropical Marine Bio-Resources and Ecology (LMB), South China Sea Institute of Oceanology, Chinese Academy of Sciences, Guangzhou 510300, China; liubing19@mails.ucas.ac.cn (B.L.); chenhuizhen20@mails.ucas.ac.cn (H.C.); shengkai23@mails.ucas.ac.cn (K.S.); fangjianxiang18@mails.ucas.ac.cn (J.F.); zhangy@scsio.ac.cn (Y.Z.); 2 College of Earth and Planetary Sciences, University of Chinese Academy of Sciences, Beijing 101408, China; 3 Xisha Marine Environmental National Observation and Research Station, Sansha 573199, China

**Keywords:** *Vibrio alginolyticus*, CsrA, central carbon metabolism, amino acid metabolism, virulence, transcription factor

## Abstract

*Vibrio alginolyticus*, a common Gram-negative opportunistic pathogen of marine animals and humans, is known for its rapid growth in organic-matter-rich environments. However, it remains unclear how it incorporates metabolic pathways in response to diverse carbon and nitrogen sources and rapidly alters gene expression. Increasing evidence suggests that post-transcriptional regulation by RNA-binding proteins and small RNAs (sRNAs) plays a crucial role in bacterial adaptation and metabolism. CsrA (carbon storage regulator A), a conserved post-transcriptional regulator in *Gammaproteobacteria*, is poorly characterized in *Vibrio* species. Using integrated transcriptomic and proteomic analyses, we found that CsrA alters the expression of 661 transcripts and 765 protein transcripts in *V. alginolyticus*, influencing key pathways including central carbon metabolism, amino acid metabolism and transport, quorum sensing, and bacterial secretion systems. Through directed CsrA-RNA EMSAs, we identified several direct mRNA targets of CsrA, including *gltB*, *gcvP*, *aceE*, and *tdh*, as well as secretion system components (*tagH*, *tssL*, *yopD*, and *sctC*). Notably, CsrA also directly regulates *rraA*, a key modulator of ribonuclease activity, suggesting a broader role in RNA metabolism. Our findings establish CsrA as a global regulator in *V. alginolyticus*, expanding the known targets of CsrA and providing new insights into its regulatory roles.

## 1. Introduction

*Vibrio* is known as the most abundant culturable bacteria in coastal water, likely due to their fast-growing ability [1]. For example, *Vibrio natriegens* is one of the fastest-growing non-pathogenic bacteria known, whose cell replication time (doubling time) can be as short as ~10 min under optimal conditions [1]. Similarly, *V. alginolyticus* is also a rapidly growing species within this genus [2], suggesting its highly efficient and flexible metabolism capability, and it can adapt to changing environments through a complex gene regulatory network. However, the way it incorporates metabolic pathways in response to diverse carbon and nitrogen sources remains unclear.

More and more studies have shown that the post-transcriptional regulatory mechanism, represented by RNA-binding proteins and non-coding small RNA (ncRNA and sRNA), plays important roles in bacterial activities [3,4]. Among these is the carbon storage regulator (Csr) (or its homolog repressor of stationary phase metabolites (Rsm)) system, which is widely distributed. It is one of the most studied post-transcriptional regulatory systems in *Gammaproteobacteria* [5]. Previous studies have established its global regulatory roles in carbon metabolism, virulence, motility, surface properties, iron homeostasis, quorum sensing, and stress responses [6,7,8,9,10,11,12,13,14,15]. Recent integrated transcriptomic studies demonstrated that the Csr system controls the expression of hundreds of genes in *Escherichia coli*, including numerous regulators [16,17,18].

CsrA, the central component of the carbon storage regulator system, functions as a homodimer protein with two symmetrical RNA-binding surfaces capable of binding to two sites within the same target RNA [19,20]. The binding site contains a conserved GGA motif that is typically presented in the loop of a short hairpin or other single-stranded regions [21]. CsrA activity is redundantly regulated by two small RNA (sRNA) antagonists, CsrB and CsrC, in *E. coli*. The high-affinity interaction of CsrA with these sRNAs outcompetes lower-affinity binding to target mRNAs by sequestering free CsrA dimers [22,23,24]. Pourciau et al. (2020) summarized the diverse mechanisms applied by CsrA of *E. coli* [5]. It often binds to the 5′-untranslated region (5′-UTR) of target mRNAs, either positively or negatively affecting RNA stability, transcript elongation, and/or the efficiency of translation [5].

More and more studies have revealed substantial structural and functional variations in the Csr system across different bacteria. For instance, some members of the *Pseudomonas* genus encode multiple CsrA homologs [25], while CsrD, a protein essential for CsrB/CsrC turnover in *E. coli*, is found only in the families *Enterobacteriaceae*, *Shewanellaceae*, and *Vibrionaceae* [25]. This diversity suggests that bacterial Csr systems have evolved to adapt to specific ecological niches. Although CsrA has been extensively studied in model organisms such as *Escherichia coli*, most studies have mainly focused on its role in carbon metabolism and have heavily relied on transcriptomic analyses to assess its global impact. However, as a post-transcriptional regulator, CsrA can significantly influence protein levels without necessarily altering mRNA abundance. Consequently, relying solely on transcriptomic approaches may underestimate its true regulatory scope. In the *Vibrionaceae* family, only a limited number of direct CsrA targets have been experimentally validated.

A transcriptomic analysis in *Vibrio cholerae* has shown that CsrA mediates processes including amino acid transport and metabolism, central metabolism, lipid metabolism, iron uptake, and motility [26]. However, this study likely overlooked many differentially expressed proteins that do not exhibit changes at the mRNA level, leading to an incomplete view of CsrA’s regulatory network. Our previous work characterized CsrA as a positive regulator of swarming motility in *V. alginolyticus* and highlighted its crucial role in metabolism, especially in amino acid metabolism [27]. Nevertheless, the broader CsrA-dependent regulatory network in *V. alginolyticus* remains largely unknown. In this study, we comprehensively investigate the CsrA regulon during early exponential-phase growth in rich medium by integrating RNA-seq and proteomics. Our findings underscore the significance of CsrA in metabolic regulation and environmental adaptation in this marine pathogen while expanding the catalog of its direct regulatory targets.

## 2. Materials and Methods

### 2.1. Bacterial Strains and Culture Conditions

All bacterial strains and plasmids used in this study are listed in Table 1. All strains were maintained at −80 °C in Luria–Bertani (LB) broth (VWR International, LLC, Radnor, PA, USA) plus 25% (*v*/*v*) glycerol. *V. alginolyticus* and derivatives were routinely cultured in Luria–Bertani (LB) broth plus 2.5% NaCl (LBS) at 30 °C. *E. coli* strains were cultured in LB medium supplemented with appropriate antibiotics at 37 °C. The culture conditions for selecting transconjugants and strains that have undergone allelic exchange were consistent with ref. [27]. For the RNA sequencing and proteomics experiment, LBS cultures from single colonies were grown overnight, diluted to a ratio of 1:1000 in LBS medium, and grown to the early exponential phase (OD_600nm_ = 0.5~0.6). For the dual-plasmid system experiment, LB cultures with appropriate antibiotics (20 μg/mL Cm and/or 100 mg/mL Amp) from single colonies were grown overnight and then diluted to a ratio of 1:300 in the same medium. Then, cultures (100 µL/well, 4 replicates in each case) were incubated at 30 °C with continuous shaking at 200 rpm in black 96-well plates with a transparent bottom. The following antibiotics were used at the indicated concentrations: for *E. coli*, 50 mg/mL kanamycin, 100 mg/mL ampicillin, and 20 mg/mL chloramphenicol; for *V. alginolyticus*, 5 mg/mL chloramphenicol. When necessary, diaminopimelate (DAP) was added to the growth media for GEB883 at a final concentration of 0.3 mM.

### 2.2. Whole-Genome RNA-Sequencing

The isolation of *V. alginolyticus* RNA, library construction and sequencing, functional annotation, and DEG analysis were performed as previously described [29]. In brief, approximately 10^9^ bacterial cells in the early logarithmic phase (OD_600nm_ = 0.5~0.6), grown in LBS medium, were pelleted by centrifugation. The experiment was conducted with three replicates. Total RNA was extracted using the TRIzol-based method (Life Technologies, CA, USA). Sequencing was performed using the Illumina Novaseq 6000 platform with pair-end 150-base reads. Genedenovo Biotechnology Co., Ltd. (Guangzhou, China) sequenced the cDNA libraries on the Illumina sequencing platform. Raw data filter and mapping were described in ref. [29]. The gene expression level was further normalized by using the fragments per kilobase of transcript per million (FPKM) mapped reads method to eliminate the influence of different gene lengths and the amount of sequencing data on the calculation of gene expression. Differentially expressed genes (DEGs) were identified using the edge R package (http://www.r-project.org/, version 3.34.0, accessed on 26 July 2021) across samples with fold changes ≥ 2 and a false discovery rate-adjusted *p* (q value) < 0.05. DEGs were then subjected to an enrichment analysis of GO function and KEGG pathways, and q values < 0.05 were used as a threshold. All RNA sequencing data are deposited in the GenBank (wildtype biosample: SAMN29675353, SRA: SRR20124473-SRR20124475; *csrA* mutant strains biosample: SAMN4356113, SRA: SRR30591842-SRR30591844) (accessed on 9 September 2024).

### 2.3. DIA-Seq and Analysis

Samples were collected using the RNA-seq method and then transferred into lysis buffer (2% SDS, 7M urea, 1 mg/mL protease inhibitor cocktail) and homogenized for 5 min on ice using an ultrasonic homogenizer. Protein extraction and protein digestion, high PH reverse phase separation, DIA (Nano-HPLC-MS/MS Analysis), protein functional annotation, and enrichment analysis were performed as previously described [29]. The experiment was conducted with three replicates. All proteome data are deposited in the iProX (Integrated Proteome Resources) database (https://www.iprox.cn/page/home.html, accessed on 19 July 2022), and the accession ID is PXD045577.

### 2.4. Generating cDNA and Quantitative PCR

Overnight cultures from a single colony were diluted to a ratio of 1:1000 into LB medium plus 2.5% NaCl (LBS). Cultures grew to the early log phase (OD_600nm_ = 0.5~0.6), and 1 × 10^9^ bacterial cells were collected. Total RNA isolation, reverse transcription, and quantitative PCR were performed as previously described [27]. The relative expression of genes was detected by qPCR using gene-specific primers (Table S1), and 16s rDNA was used as an internal reference as described previously [27]. Relative levels were calculated using the threshold cycle (ΔΔCT) method [30] and normalized to the wildtype ZJ-T value. Each reaction produced only one melting curve, indicating that only one target had been amplified during the qPCR reaction. Measurements were performed in triplicate. Statistical significance was determined using a one-way ANOVA followed by Tukey’s HSD test.

### 2.5. CsrA Recombinant Protein Construction and Purification

The recombinant protein construction was performed as previously described with minor modifications [29]. In brief, the full-length encoding sequence of the *csrA* gene was amplified by PCR with the primer sets CsrA-ORF_F/CsrA-ORF_R (Table S1). The plasmid pET28b was amplified with linearized primer pairs pET28b_F/R, and the fragments were then inserted into plasmid pET28b with a ClonExpress^®^ II One Step Cloning Kit (Vazyme Biotech Co., Ltd., Nanjing, China) to obtain a recombinant plasmid, which was transformed into *E. coli* BL21 (DE3)-competent cells. Purification was performed using the same protocol described in ref. [29].

### 2.6. RNA Electrophoretic Mobility Shift Assays

A biotin-labeled *rraA* probe was synthesized by Sangon Biotech (Shanghai, China), and the CsrA- *rraA* RNA EMSA samples were prepared according to the instructions for an RNA EMSA kit (BersinBio, Guangzhou, China). For other probe synthesis, the T7 High Yield RNA Transcription kit (Vazyme Biotech Co., Ltd., Nanjing, China) was used to transcribe RNA in vitro. For this method, the RNA product of the mRNA was generated using a forward primer containing the T7 promoter (Table S1). Each region that was PCR amplified includes 100 nucleotides upstream from the ATG and 100 nucleotides in the coding region. The T7 PCR product was purified using the EasyPure^®^ RNA Purification kit (TransGen Biotech, Beijing, China), and the RNA was either used immediately or placed at −80 °C until further use. The gel mobility shift assay was performed using a similar protocol version described in ref. [31] with minor modifications. The RNA was dissolved in DEPC water, heated to 80 °C for 10 min, and slowly cooled to room temperature before use. For the RNA-binding test, 1 μL of pre-treated RNA solution was incubated at 25 °C for 30 min with CsrA protein and RNA inhibitor (0.5 μL/reaction) at the indicated concentrations. Then the resultant reactions were separated using 1.5% TBE PAGE, and the gel was stained with SYBR Green II (Biosharp), and the RNA bands were visualized with Gel Doc^TM^ XR^+^ (Bio-Rad Laboratories, Inc., Hercules, CA, USA).

## 3. Results

### 3.1. Transcriptome and Proteomics Analysis of Wildtype and CsrA Mutant

To better understand the regulatory role of CsrA in *V. alginolyticus*, we used RNA-seq to compare transcriptomic changes at the early exponential phase between the *csrA* point mutant ZJ-T-*csrA*R6H and the wildtype strain, ZJ-T. The output statistics of RNA sequencing shown in Table S2 indicate good sequencing quality, suggesting that the subsequent transcriptome analysis results are reliable.

**Figure 1:**
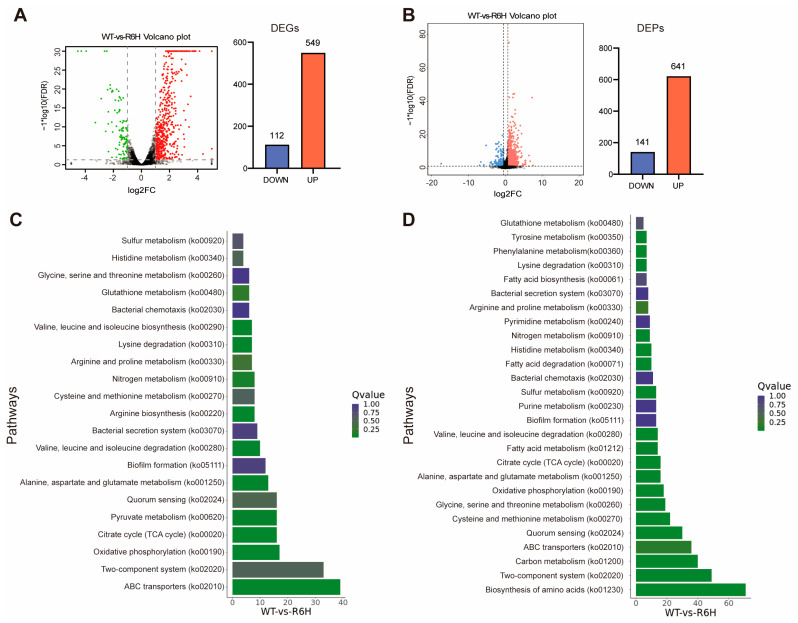
Overview of RNA transcriptomic and proteomic profiles of wildtype and *csrA* mutant strains. (**A**) Volcanic map of differential genes in transcriptome (FDR < 0.05, |log2 (fold change)| ≥ 1) (**left** panel) and statistical column chart of differentially expressed genes (**right** panel). WT: wildtype strain *V. alginolyticus* ZJ-T; R6H: *csrA* mutant strain ZJ-T-*csrA*R6H. Red dot represents significantly upregulated difference; green dot represents significantly downregulated difference; black dot represents no difference. (**B**) Volcanic map of differential proteins in proteomics (FDR < 0.05, |fold change| ≥ 1.5) (**left** panel) and statistical column chart of differentially expressed proteins (**right** panel). Red dot represents significantly upregulated difference; blue dot represents significantly downregulated difference; gray dot represents no difference. WT: wildtype strain *V. alginolyticus* ZJ-T; R6H: *csrA* mutant strain ZJ-T-*csrA*R6H. (**C**) Histogram of top 21 KEGG pathway enrichments in transcriptomics after mutation of *csrA*. (**D**) Histogram of top 26 KEGG pathway enrichments in proteomics after mutation of *csrA*.

A total of 661 differentially expressed genes (DEGs) were identified (FDR < 0.05 and |log2FC| ≥ 1) (Figure 1A and Table S3), accounting for 14.17% of the *V. alginolyticus* transcriptome. Among these DEGs, 549 were upregulated, while 112 were downregulated (Figure 1B). To investigate the biological processes and pathways affected by *csrA* mutation, a KEGG (Kyoto Encyclopedia of Genes and Genomes) pathway enrichment analysis was performed on the identified DEGs (Figure 1C). From this, we found that CsrA affects the expression of multiple categories of processes related to metabolism, including the tricarboxylic acid (TCA) cycle, amino acid synthesis and degradation, ABC transporters, and oxidative phosphorylation. These findings are similar to those reported by Butz et al. for *V. cholera* [26].

To further reveal the post-transcriptional regulatory network of CsrA, DIA (Data-independent Acquisition) quantitative proteomics was performed to compare the proteomics changes between *V. alginolyticus* ZJ-T and ZJ-T-*csrA*R6H. A total of 762 differential expression proteins (DEPs) (FDR < 0.05 and |FC| ≥ 1.5) were identified following the *csrA* mutation, of which 621 showed increased abundance. In contrast, 141 showed decreased abundance (Figure 1B). KEGG pathway mapping of all DEPs revealed widespread differential regulation of genes involved in carbohydrate and amino acid metabolism. Among the most enriched pathways, amino acid biosynthesis and metabolism, the TCA cycle, and fatty acid metabolism are the three most represented pathways (Figure 1D), followed by pathways related to the ABC transporter, two-component system, quorum sensing, oxidative phosphorylation, and other essential biological processes.

**Figure 2:**
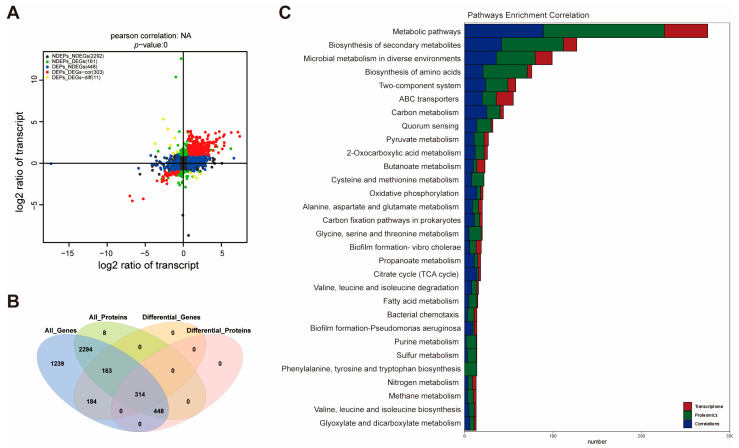
Integrated analyses of proteomics and transcriptomics. (**A**) Four-quadrant map analysis of proteomics and transcriptomics data. NDEPs, Non-Differentially Expressed Proteins; NDEGs, Non-Differentially Expressed Genes; DEGs, Differentially Expressed Genes; DEPs, Differentially Expressed Proteins. (**B**) Venn diagram of differentially expressed genes (DEGs) and differentially expressed proteins (DEPs) in wildtype and *csrA* mutant strains. (**C**) Pathway enrichment and correlation in wildtype and *csrA* mutant strains.

To gain a comprehensive understanding of the regulatory impact of *csrA* on gene expression, we performed an integrated analysis of transcriptomic and proteomic data. A total of 661 differentially expressed genes (DEGs) and 762 differentially expressed proteins (DEPs) (Figure 1A,D) account for 14.17% of the *V. alginolyticus* transcriptome and 23.6% of the proteome, respectively. To visualize the correlation between mRNA and protein expression changes, a four-quadrant map was constructed based on transcriptomic and proteomic data (Figure 2A), providing insights into the post-transcriptional regulatory mechanisms mediated by *csrA*. Among all annotated genes, 2294 showed no differential expression at either the transcriptomic or proteomic level. In contrast, 314 genes exhibited differential expression in both datasets. A total of 163 genes were differentially expressed only at the transcriptomic level. At the same time, 448 displayed differential expression exclusively at the proteomic level, suggesting that their regulation by CsrA likely occurs post-transcriptionally, influencing protein abundance independently of mRNA expression (Figure 2B).

Notably, as shown in Figure 2C, the enriched pathways identified in the transcriptomic and proteomic datasets do not exhibit a strong correlation. In general, the number of differentially expressed proteins detected in the proteomic dataset is lower than the number of differentially expressed genes in the transcriptomic dataset, further supporting the post-transcriptional regulatory role of CsrA. For instance, in the “Biosynthesis of amino acids” pathway, 24 genes and 71 proteins were annotated to this pathway, among which 21 showed differential expression at both the transcriptomic and proteomic levels. A similar trend is observed across most pathways. However, exceptions exist, such as the TCA cycle, where the number of differentially expressed genes closely matches that of differentially expressed proteins, indicating a strong correlation. This suggests that CsrA may exert direct transcription regulation in addition to its well-established post-transcriptional control, thereby influencing protein expression at multiple levels. Overall, the correlation between the transcriptomic and proteomic data highlights that CsrA employs distinct regulatory mechanisms across different targets and pathways, exerting both transcriptional and post-transcriptional control to modulate gene expression.

### 3.2. Profiles of CsrA Regulatory Mode

#### 3.2.1. The Role of CsrA in Central Carbon and Energy Metabolism

CsrA has been known to regulate central carbon metabolism since the 1990s [7]. Previous studies have confirmed that CsrA activates glycolysis, while it represses gluconeogenesis and the TCA cycle [7,17,32]. Moreover, the mutation of *csrA* results in coordinated changes in the expression of numerous genes in glycolysis, gluconeogenesis, and the TCA cycle. It suggests that CsrA may also indirectly regulate central carbon metabolism genes [17]. Here, we summarize the roles of CsrA in carbon metabolism and discuss them in categories based on the pathways involved.

##### Glycogen Biosynthesis, Glycolysis, and Gluconeogenesis

The expression profiles of GAPDH, PdhA, PdhB, Fbp, and GlgA were selected. GlgA (glycogen synthase) was upregulated by 1.77-fold in the proteome of ZJ_T-*csrA*R6H, suggesting that CsrA may inhibit glycogen biosynthesis. In addition, compared with ZJ-T, Fbp (fructose-1,6-bisphosphatase), acting as a rate-limiting enzyme in gluconeogenesis, was upregulated by approximately 2-fold both in transcriptomics and proteomics. In addition, GAPDH (glyceraldehyde-3-phosphate dehydrogenase), a key enzyme in glycolysis and gluconeogenesis that catalyzes the first step of the pathway by converting D-glyceraldehyde 3-phosphate (G3P) into 3-phospho-D-glyceroyl phosphate reversibly, was upregulated by 1.84-fold in proteomics. This suggests that CsrA may inhibit glycogen biosynthesis, glycolysis, and gluconeogenesis.

##### Pyruvate and TCA Cycles

Pyruvate dehydrogenase (PDH) is a key regulatory node in the metabolic fine-tuning between glucose and fatty acid oxidation. The *V. alginolyticus* genome encodes two isoenzymes of the pyruvate dehydrogenase complex, *pdhAB* and *aceEF*. The mutation of *csrA* specifically affected the expression of *pdhAB* but had no impact on *aceEF* expression. This result differs from the finding in *E. coli*, where CsrA affects the RNA abundance and translation of AceEF [17]. This difference may be attributed to the absence of the *pdhAB* isoenzyme in *E. coli* as these proteins are predominantly found in Gram-positive bacteria such as *Mycobacterium*. Interestingly, the *pdhAB*-encoded complex is a secreted protein, unlike *aceEF*, which encodes a cytoplasmic protein. This distinction may further contribute to the differential regulation of these isoenzymes by CsrA in *V. alginolyticus* compared to *E. coli*.

**Figure 3:**
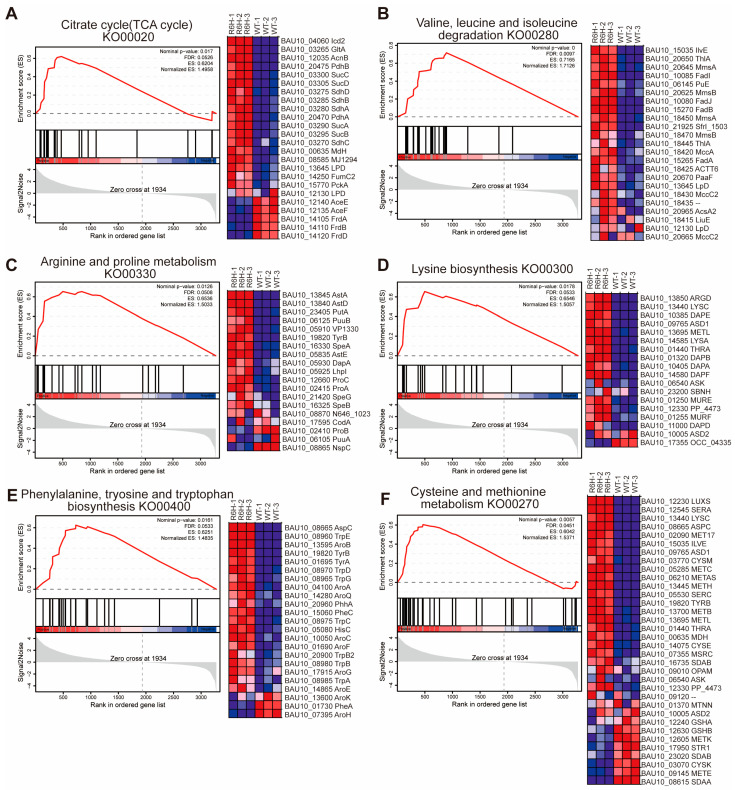
Gene Set Enrichment Analysis (GSEA) and gene expression heatmap of key pathways in TCA cycle and amino acid metabolism. (**A**) (**left** panel) GSEA of TCA cycle pathway in *csrA* mutant compared to wildtype. Normalized enrichment score (NES) and false discovery rate (FDR) are indicated. Significant enrichment (FDR = 0.05) suggests altered TCA cycle activity in *csrA* mutant. (**right** panel) Heatmap of gene expression levels for key genes involved in TCA cycle. (**B**) GSEA of valine, leucine, and isoleucine degradation pathway in *csrA* mutant compared to wildtype (FDR < 0.05) and heatmap of gene expression levels for key genes involved in this pathway. (**C**) GSEA of arginine and proline metabolism pathway in *csrA* mutant compared to wildtype (FDR = 0.05) and heatmap of gene expression levels for key genes involved in this pathway. (**D**) GSEA of lysine biosynthesis pathway in *csrA* mutant compared to wildtype (FDR = 0.05) and heatmap of gene expression levels for key genes involved in this pathway. (**E**) GSEA of phenylalanine, tryosine, and tryptophan biosynthesis pathway in *csrA* mutant compared to wildtype (FDR, = 0.05) and heatmap of gene expression levels for key genes involved in this pathway. (**F**) GSEA of cysteine and methionine metabolism pathway in *csrA* mutant compared to wildtype (FDR = 0.05) and heatmap of gene expression levels for key genes involved in this pathway. Rows represent genes, and columns represent samples. Expression levels are normalized and color-coded (red: upregulation; blue: downregulation). Data are derived from RNA-seq analysis (*n* = 3).

Here, we found that PDHA and PDHB are upregulated by 2.40-fold and 3.79-fold in proteomics, and the transcript of *pdhA* was upregulated by 4.61-fold. Hence, this implies that CsrA may negatively regulate the reaction from PEP to pyruvate, leading to the restriction of carbon flux into the TCA cycle. The Gene Set Enrichment Analysis (GSEA) results demonstrate the significant enrichment of TCA cycle-related genes in the experimental group (NES = 1.50, *p* = 0.017, FDR = 0.05). The enrichment score (ES) curve (Figure 3A) shows that the peak ES value is located at the top-ranked genes, indicating the upregulation of the TCA cycle pathway in the *csrA* mutant. Furthermore, a leading edge analysis identified key contributors to this enrichment (Figure 3A). The genes *mdh* (malate dehydrogenase), *icd2* (isocitrate dehydrogenase), *cs* (citrate synthase), *acnB* (aconitate hydratase 2/2-methylisocitrate dehydratase), *sucA* (2-oxoglutarate dehydrogenase E1 component), *sucB* (2-oxoglutarate dehydrogenase E2 component), *sucC* (succinyl-CoA synthetase beta subunit), *sucD* (succinyl-CoA synthetase alpha subunit), *sdhA* (succinate dehydrogenase/fumarate reductase, flavoprotein subunit), and *sdhB* (succinate dehydrogenase/fumarate reductase, iron-sulfur subunit), which are responsible for the TCA cycle, are all upregulated in ZJ-T-*csrA*R6H by 2~7-fold, and the protein production of these genes is also increased by 1.97~5.88-fold compared with ZJ-T. These results indicate that CsrA negatively regulates the pyruvate and TCA cycles at multiple points.

##### Oxidative Phosphorylation

Genes involved in oxidative phosphorylation are significantly enriched in our transcriptomics and proteomics datasets. Notably, CsrA represses the expression of key genes and their corresponding proteins involved in electron transport and energy metabolism, including cytochrome c oxidase cbb3-type (*ccoNOPQ*), ubiquinol-cytochrome c reductase (*petABC*), fumarate reductase (*frdABC*), succinate dehydrogenase (*sdhABC*), and cytochrome o ubiquinol oxidoreductase (*cyoAB*) (Table S3). The mutation of *csrA* leads to a substantial upregulation of the oxidative phosphorylation system, suggesting a potential role in enhancing ATP synthesis and possibly influencing bacterial growth. Cytochromes (*cyts*), for instance, are ubiquitous heme-containing proteins that serve as key components of energy transduction pathways, including photosynthesis and respiration. They participate in a wide variety of electron transfer reactions, which are crucial for ATP production and overall cellular energy homeostasis [33]. Overall, CsrA regulates the expression of more than 20 genes encoding components of the oxidative phosphorylation system and other oxidoreductases (Table S3), underscoring the critical role of CsrA in modulating ATP synthesis and energy metabolism *in V. alginolyticus*.

#### 3.2.2. The Role of CsrA in Amino Acid Metabolism and ABC Transporters

##### Amino Acid Metabolism

**Figure 4:**
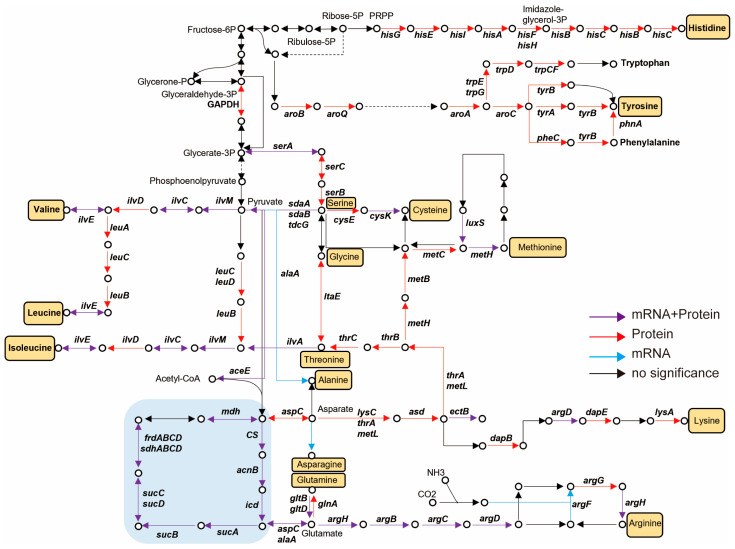
CsrA regulates the tricarboxylic acid cycle and amino acid metabolism. The frame filled with yellow represents different pathways of amino acids. The red arrow represents differential expression only in the proteome, the purple arrow represents differential expression in both the transcriptome and proteome, and the blue arrows represent differential expression only in the transcriptome. The black arrows indicate no difference.

Overall, CsrA regulates the expression of over 100 genes and/or proteins involved in amino acid metabolism. These include pathways such as: alanine, aspartate, and glutamate metabolism; valine, leucine, and isoleucine biosynthesis and degradation (Figure 3B); arginine and proline metabolism (Figure 3C); lysine biosynthesis (Figure 3D); phenylalanine, tyrosine, and tryotophan biosynthesis (Figure 3E); histidine metabolism; and cysteine and methionine metabolism (Figure 3F). The GSEA results indicate that CsrA broadly represses amino acid metabolism during the early exponential phase, a critical period for nutrient and energy allocation. The regulatory roles of CsrA identified in the TCA cycle and amino acid metabolism are summarized in Figure 4, where the relevant genes involved in these pathways are mapped within the metabolic network.

For example, *gltB* (BAU10_01395) and *gltD* (BAU10_01390), which encode glutamate synthase, a key enzyme in amino acid biosynthesis, are upregulated by over 10-fold in ZJ-T-*csrA*R6H. Their productions are upregulated by over 70-fold in the *csrA* mutant strain, indicating a significant regulatory effect of CsrA on glutamate metabolism. A notable pathway identified in our analysis was branched-chain amino acid (BCAA) metabolism. In bacteria, four main operons are known to regulate BCAA biosynthesis, namely *ilvIH*, *ilvBN*, *ilvGMEDA*, and *leuABCD*, respectively [34,35,36,37]. In ZJ-T-*csrA*R6H, the expression levels of genes involved in BCAA biosynthesis were upregulated by approximately 2-fold compared with the wildtype strain. Furthermore, a set of genes and/or proteins involved in BCAA degradation also exhibited increased expression, including MmsB (3-hydroxyisobutyrate dehydrogenase), MmsA (malonate-semialdehyde dehydrogenase (acetylating)), IVD (isovaleryl-CoA dehydrogenase), FadB (3-hydroxyacyl-CoA dehydrogenase), FadJ (3-hydroxyacyl-CoA dehydrogenase), MccA (3-methylcrotonyl-CoA carboxylase alpha subunit), and Mccc2 (3-methylcrotonyl-CoA carboxylase beta subunit). This pattern of expression shows that CsrA represses BCAA biosynthesis and degradation at the early exponential phase. This expression pattern indicates that CsrA acts as a global repressor of both BCAA biosynthesis and degradation during the early exponential phase.

Additionally, 10 differentially expressed genes and/or proteins (HisD, HisG, HisC, HisB, HisI, HisA, HisF1, HisH, HutG, and HutU) are enriched in histidine metabolism. These genes are known to be involved in histidine synthesis from phosphoribosyl pyrophosphate (PRPP) (major pathway) and histidine catabolism to glutamine (minor pathway). The mutation of *csrA* resulted in a marked upregulation of both histidine synthesis and degradation, further highlighting its role in amino acid metabolism.

##### ABC Transporters

**Figure 5:**
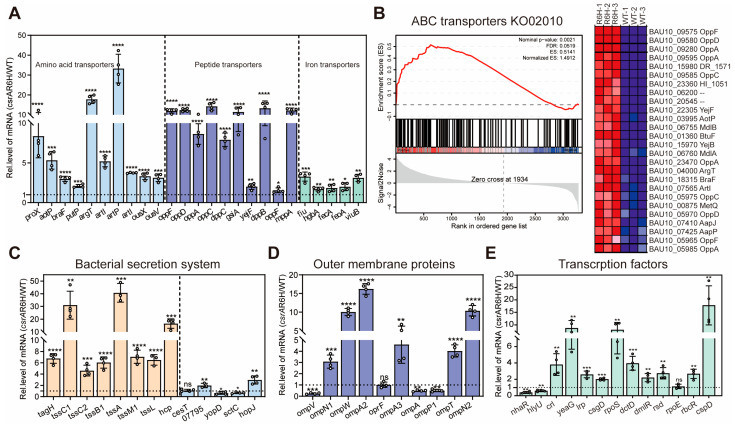
CsrA modulates genes involved in ABC transporters, the bacterial secretion system, the outer membrane, and transcription factors. (**A**) The expression of genes of amino acid transporters, peptide transporters, and siderophore-related genes by qPCR in wildtype and *csrA* mutant strains. Blue represents genes of amino acid transport (*proX*, *aotP*, *braF*, *putP*, *argT*, *artI*, *artP*, *artI*, *ousX*, and *ousV*), purple represents genes of oligopeptide transport (*oppF*, *oppD*, *oppA*, *oppC*, *dppC*, *gsiA*, *yejF*, *dppB*, *oppF*, and *mppA*), and green represents genes of iron transport (*fiu*, *hgbA*, *fecA*, *feoA*, and *viuB)*. (**B**) (**left** panel) The GSEA of the ABC transporter pathway in the *csrA* mutant compared to the wildtype. The normalized enrichment score (NES) and false discovery rate (FDR) are indicated. Significant enrichment (FDR = 0.05) suggests an altered ABC transporter pathway in the *csrA* mutant. (**right** panel) A heatmap of the gene expression levels for selected key genes involved in the ABC transporter. Rows represent genes, and columns represent samples. Expression levels are normalized and color-coded (red: upregulation; blue: downregulation). Data are derived from RNA-seq analysis (*n* = 3). (**C**) The expression of genes involved in bacterial secretion systems in wildtype and *csrA* mutant strains by qPCR. Orange represents genes involved in T6SS (*tagH*, *tssC1*, *tssC2*, *tssB1*, *tssA*, *tssL*, *tssM1*, and *hcp*). Blue represents genes involved in T3SS (*cesT*, *BAU10_07795*, *yopD*, *sctC*, and *hopJ*). (**D**) The expression of genes involved in outer membrane proteins (*ompV*, *ompN1*, *ompW*, *ompA2*, *oprF*, *ompA3*, *ompA*, *ompP1*, *ompT*, and *ompN2*) in the wildtype and *csrA* mutant strains by qPCR. (**E**) The expression of genes involved in outer transcription (*nhaR*, *hlyU*, *crl*, *yeaG*, *lrp*, *csgD*, *rpoS*, *dctD*, *dmlR*, *rsd*, *rpoE*, *rbcR*, and *cspD*) in the wildtype and *csrA* mutant strains by qPCR. The relative expression of the ZJ-T-*csrA*R6H strain is divided by that of the WT. The data are presented as the mean ± SD. Statistical significance was determined using Student’s *t*-test, *p* value: ns, *p* > 0.05; *, *p* < 0.05; **, *p* < 0.01; ***, *p* < 0.001; ****, *p* < 0.0001.

Amino acids are essential substances for protein and nucleic acid synthesis, but amino acids and oligopeptides need to be taken up into the cell by specific amino acid transporters on the cell membrane before they are available for the cellular process. Previous studies showed that the amino acid utilization of ZJ-T-*csrA*R6H is attenuated compared with the wildtype strain, especially L-proline, in which the *csrA* mutant strain is not able to grow on M63 minimal medium supplemented with L-proline [27]. qPCR was performed to validate the expression of amino acid transporter-associated genes (*proX*, *aotP*, *braF*, *putP*, *argT*, *artI*, *artP*, *artI*, *ousX*, and *ousV*), peptide transporter-associated genes (*oppF*, *oppD*, *oppA*, *oppC*, *dppC*, *gsiA*, *yejF*, *dppB*, *oppF*, and *mppA*), and iron transporter-associated genes (*fiu*, *hgbA*, *fecA*, *feoA*, *viuB*) in wildtype and *csrA* mutant strains (Figure 5A). The qPCR results and GSEA (Figure 5B) show that, to a large extent, CsrA represses amino acid and oligopeptide transporters by negatively affecting the transcript abundance of the genes related to amino acid and oligopeptide transporter biosynthesis. For instance, *aapP* and *aapJ*, which are required for the general L-amino acid transport system ATP-binding protein, are upregulated by more than 2-fold (FDR < 0.05) both in the transcriptome and proteome.

There are always exceptions, such as artP and artI, which are involved in the arginine transport system ATP-binding proteins are upregulated by more than 4-fold (FDR < 0.05) in transcriptome but show decreased trends in proteome, suggesting that CsrA regulates *artP* and *artI* post-transcriptionally. Taken together, these results may suggest that CsrA regulates ABC transporters by affecting the transcript abundance of the genes related to transporters and/or in a post-transcriptional way in *V. alginolyticus.*

In addition, we identified genes encoding several inorganic iron and ferrous iron uptake systems, including those for TonB-dependent siderophore receptors (*fiu*, *hgbA*, *fct*, and *fecA*), as well as *feoA* (encoding ferrous iron transport protein A) and *viuB* (encoding siderophore utilization protein). The qPCR results reveal that *fiu*, *hgbA*, *fecA*, *feoA*, and viuB were all significantly upregulated in the *csrA* mutant (Figure 5A). However, in the proteomics analysis, we found that FeoA was downregulated, and ViuB exhibited no significant difference; the other proteins were upregulated in the *csrA* mutant. To colonize iron-restricted environments within the host and fulfill their nutritional requirements, bacteria have evolved diverse siderophore-mediated iron acquisition mechanisms. In Gram-negative bacteria, TonB-dependent receptors facilitate the transport of siderophores into the periplasm [38]. The observed upregulation of four TonB-dependent receptors in the *csrA* mutant suggests that CsrA plays a crucial role in restricting the influx of free, unchelated iron during the early exponential phase while simultaneously regulating intracellular iron homeostasis. Collectively, these findings highlight the importance of CsrA in the regulation of amino acid, oligopeptide, and iron transport, further underscoring its role in global metabolic control.

#### 3.2.3. The Role of CsrA in Virulence

##### Bacterial Secretion Systems

Bacterial secretion systems are macromolecular complexes that facilitate the release of virulence factors into the extracellular environment or directly translocate them into the target host cell, playing a crucial role in pathogenesis [39]. The Type III secretion system (T3SS) is widely distributed among Gram-negative bacteria [40] and is essential for their survival and replication within the host cells [40]. The Type VI secretion system (T6SS) is widespread in both pathogenic and non-pathogenic bacteria and has been implicated in interspecies competition [41,42,43].

To investigate the regulatory role of CsrA in these secretion systems, we screened differentially expressed T3SS- and T6SS-related genes and proteins through transcriptomic and proteomic analyses, with further validation using qRT-PCR. As shown in Figure 5C and Figure S3, approximately three-quarters of T3SS genes were downregulated at the protein level in the *csrA* mutant despite there being no significant changes at the transcriptional level. This suggests that CsrA post-transcriptionally activates T3SS in *V. alginolyticus*, consistent with findings in other bacterial species [44,45]. Interestingly, the impairment of CsrA resulted in the upregulation of CesT (BAU10_07875), an effector chaperone known to antagonize CsrA activity through direct protein interaction [31]. This observation suggests that CsrA represses CesT translation in *V. alginolyticus*, potentially establishing a reciprocal feedback regulatory loop between T3SS and CsrA.

Additionally, the homologs of YopD and LcrH, which function as post-transcriptional repressors of T3SS in *Yersinia pestis* [46], were found to be downregulated by 6- and 10-fold, respectively, in the mutant. This suggests that CsrA enhances their expression. In contrast, the impairment of CsrA resulted in the increased expression of T6SS both at the transcriptomic and proteomic levels, indicating that CsrA negatively regulates T6SS of *V. alginolyticus*.

##### Outer Membrane Proteins

Outer membrane β-barrel proteins (OMPs) play a crucial role in bacterial physiology, contributing to membrane integrity, nutrient uptake, and interactions with the environment. In the *csrA* mutant, the majority of OMP-encoding genes, including *ompN1*, *ompW*, *ompA2*, *ompA3*, *ompT*, and *ompN2*, were significantly upregulated, whereas the second copies of *ompA* and *ompP1* were downregulated (Table S3). These transcriptional changes were further validated by qPCR (Figure 5D), which confirmed the significant upregulation of *ompN1*, *ompW*, *ompA2*, *ompA3*, *ompT*, and *ompN2*, as well as the downregulation of *ompA* and *ompP1*, consistent with the transcriptomic data. Although discrepancies were initially observed in *ompV* expression between the transcriptomic and qPCR results, further analysis revealed that the transcriptomic fold change (−1.32) did not meet the threshold for significance but still reflected the same downregulation trend observed in the qPCR data. These findings suggest that alterations in CsrA levels remodel the outer membrane composition of *V. alginolyticus*.

To further explore this possibility, a proteomics analysis revealed that, with the exception of OmpA and OmpP1, which exhibited a significant decrease, all other OMPs were significantly upregulated, particularly OmpT, which showed a more than 6-fold increase in expression. This indicates that CsrA regulates the expression of OMP-related genes, leading to the increased biosynthesis of most OMPs. However, the repression of OmpA and OmpP1 in the *csrA* mutant suggests that CsrA exerts differential regulatory effects on distinct OMPs, further reinforcing its role in outer membrane remodeling in *V. alginolyticus*.

##### Quorum Sensing

CsrA is well-established as a key regulator of quorum sensing (QS), a mechanism that enables bacteria to synchronize gene expression in response to population density. In the *csrA* mutant, not surprisingly, a significant number of QS-related genes exhibited altered expressions, including *luxS*, *luxP*, *cqsS*, *cqsA*, *luxO*, *luxU*, and *aphA*. Notably, *luxP*, *cqsS*, *cqsA*, and *luxU* were identified as previously unrecognized targets of CsrA regulation, expanding their known role in QS control. Regulatory patterns suggest that CsrA positively regulates *luxP* and *aphA* but regulates *luxS*, *cqsS*, *cqsA*, *luxO*, and *luxU* in a negative manner. These findings indicate that CsrA plays a more intricate role in quorum sensing than previously reported, further highlighting its importance in global gene regulation.

#### 3.2.4. CsrA: A Regulator of Regulators

RNA-seq and proteomic analyses identified several transcriptional regulators as notable targets of CsrA-mediated regulation. These include global regulators, such as two-component systems, sigma factors, and cold shock proteins; and local factors, such as DmlR (involved in C4-dicarboxylates metabolism) and Lrp (leucine-responsive regulatory protein). Among the global regulators, Crl, a transcriptional factor known to enhance RpoS activity [47], was significantly upregulated in the *csrA* mutant (6.91-fold in proteomics; 3.76-fold in transcriptomics). Similarly, the response regulator CsgD (5.38-fold in proteomics; 4.37-fold in transcriptomics), sigma factors RpoS (3.2-fold in proteomics; 2.01-fold in transcriptomics), and RpoE (1.54-fold in proteomics) were all upregulated. Notably, CspD, a cold shock-like protein, exhibited the most dramatic increase (165.3-fold in proteomics; 9.36-fold in transcriptomics) (Table S3). These transcriptional changes were further confirmed by qPCR analysis (Figure 5E).

Cold shock proteins play critical roles in transcriptional antitermination, transcript stability, and translation initiation in *E. coli* [48,49]. In *Bacillus subtilis*, CspB and CspD have been reported to influence approximately 20% of gene expression in *Bacillus subtilis* [50]. In this study, we observed that *cspD* transcript levels increased by 9.4-fold, while its protein levels increased by 165.4-fold in the *csrA* mutant, suggesting that CsrA exerts substantial post-transcriptional control over cold shock proteins.

In addition to global regulators, local transcription factors were also significantly upregulated in the *csrA* mutant, including the LysR family transcriptional regulators DmlR and RbcR (5.52-fold and 5.34-fold, respectively) and Lrp (5.30-fold in proteomics; 2.09-fold in transcriptomics). This regulatory pattern suggests that CsrA broadly represses transcriptional regulators during the early exponential phase, leading to a general dampening of global gene expression and a tightly controlled regulatory network.

### 3.3. CsrA Represses the Translation of Rraa by Direct Binding

Previous studies have shown that *csrA* impairment alters the stability of numerous mRNA candidates in *V. alginolyticus* [27], a phenomenon also reported in other bacteria. Here, no significant changes were observed in the major components of the RNA degradosome in response to *csrA* mutation. However, *rraA* (*regulator of ribonuclease activity A*), a known RNase E binding protein, was significantly upregulated in the *csrA* mutant by 5.0-fold at the transcript level and by 9.9-fold at the protein level (Table S3). RraA acts as a protein inhibitor of RNase E by directly interacting with and inhibiting its endonucleolytic cleavage activity [51]; thus, it is regarded as a key regulator of RNase E function. These findings suggest a potential link between CsrA and RNase E activity through RraA regulation.

**Figure 6:**
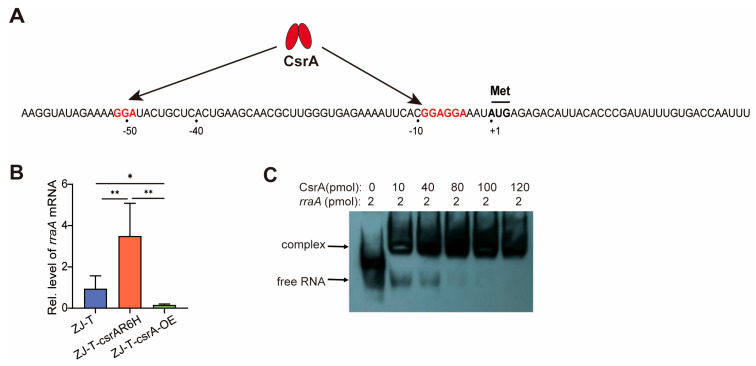
CsrA represses the translation of RraA by direct binding. (**A**) A schematic representation of the 5′ untranslated region (5′UTR) of *rraA*. The transcription start site (+1) and the translation start codon (AUG) are indicated. The 5′UTR contains three GGA motifs, which are putative CsrA binding sites; two of these motifs are located adjacent to the ribosome binding site (RBS). All thymine (T) bases have been replaced with uracil (U) to accurately represent the RNA sequence. (**B**) The relative expression of *rraA* in the wildtype ZJ-T, *csrA* mutant, and *csrA* overexpression strains. (Student’s *t*-test, *p* values: *, <0.05; **, <0.01). *rraA* levels were normalized to the level of the housekeeping gene *recA*. Error bars indicate standard deviations. (**C**) EMSA showing the 6xHis-CsrA-*rraA* RNA interaction. The RNA probe sequence is −64 to +36 for a total of 100 bp.

To further explore the regulatory relationship between CsrA and RraA, we analyzed the flanking sequences of the *rraA* translation initiation region. As shown in Figure 6A, this region contains three GGA motifs, which are potential binding sites for CsrA, and two GGA motifs located adjacent to the ribosome binding site (RBS) (Figure 6A). A 5′-RACE analysis identified at least three transcriptional start sites for *rraA*, with the longest transcript containing a 493-nt 5′-UTR [52]. Electrophoretic mobility shift assays (EMSAs) demonstrated that CsrA binds to the 5′-UTR of *rraA* with high affinity in vitro (Figure 6C), indicating direct regulation of *rraA* expression.

To assess whether *CsrA* influences *rraA* transcript abundance, we examined *rraA* expression in *csrA* overexpression strains constructed by [27]. In these strains, *csrA* expression was upregulated by 1.72-fold compared to the wildtype [27]. Using qRT-PCR, we quantified *rraA* mRNA levels in the wildtype strain (ZJ-T), the *csrA* mutant, and the *csrA* overexpression strain. As shown in Figure 6B, *rraA* mRNA levels were elevated by 4-fold in the *csrA* mutant, whereas *csrA* overexpression significantly reduced *rraA* mRNA abundance, consistent with transcriptomic data, suggesting that CsrA negatively regulates *rraA* expression.

### 3.4. RNA EMSA Identified RNA Targets That Interact with Csra

CsrA is known to bind specific GGA motif (s) within the 5′ untranslated region (5′-UTR) and/or the early coding region of its mRNA targets, leading to alterations in RNA structure, translation, stability, and/or transcription elongation [13]. To investigate direct CsrA-mediated regulation, we selected multiple target genes from distinct functional pathways and assessed their interaction with CsrA using RNA EMSA.

**Figure 7:**
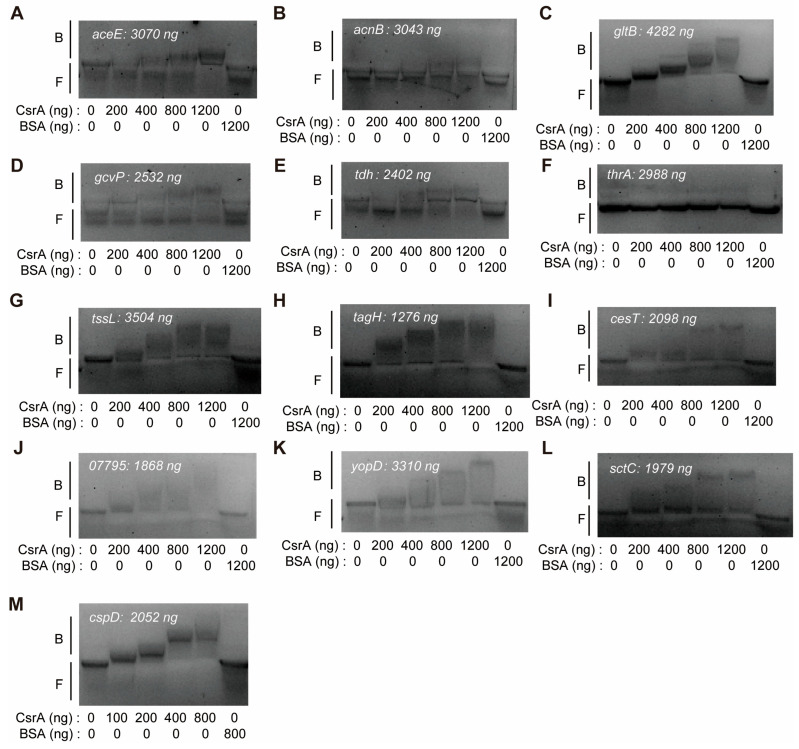
The RNA EMSA identified RNA targets that interact with CsrA. (**A**) An EMSA showing the 6xHis-CsrA-*aceE* RNA interaction (−100 to +100 nt relative to the start codon). The amount of *aceE* RNA is shown. (**B**) An EMSA showing the 6xHis-CsrA-*acnB* RNA interaction (−100 to +100 nt relative to the start codon). The amount of *acnB* RNA is shown. (**C**) An EMSA showing the 6xHis-CsrA-*gltB* RNA interaction (−100 to +100 nt relative to the start codon). The amount of *gltB* RNA is shown. (**D**) An EMSA showing the 6xHis-CsrA-*gcvP* RNA interaction (−100 to +100 nt relative to the start codon). The amount of *gcvP* RNA is shown. (**E**) An EMSA showing the 6xHis-CsrA-*tdh* RNA interaction (−100 to +100 nt relative to the start codon). The amount of *tdh* RNA is shown. (**F**) An EMSA showing no interaction between 6xHis-CsrA and *thrA* RNA (−100 to +100 nt relative to the start codon). The amount of *thrA* RNA is shown. (**G**) An EMSA showing the 6xHis-CsrA-*tssL* RNA interaction (−100 to +100 nt relative to the start codon). The amount of *tssL* RNA is shown. (**H**) An EMSA showing the 6xHis-CsrA-*tagH* RNA interaction (−100 to +100 nt relative to the start codon). The amount of *tagH* RNA is shown. (**I**) An EMSA showing the 6xHis-CsrA-*cesT* RNA interaction (−100 to +100 nt relative to the start codon). The amount of *cesT* RNA is shown. (**J**) An EMSA showing the 6xHis-CsrA-*BAU10_07795* RNA interaction (−100 to +100 nt relative to the start codon). The amount of *BAU10_07795* RNA is shown. (**K**) An EMSA showing the 6xHis-CsrA-*yopD* RNA interaction (−100 to +100 nt relative to the start codon). The amount of *yopD* RNA is shown. (**L**) An EMSA showing the 6xHis-CsrA-*sctC* RNA interaction (−100 to +100 nt relative to the start codon). The amount of *sctC* RNA is shown. (**M**) An EMSA showing the 6xHis-CsrA-*cspD* RNA interaction (−100 to +100 nt relative to the start codon). The amount of *cspD* RNA is shown.

A comprehensive analysis of GGA motifs was performed on gene sequences spanning positions −100 to +100 relative to the translation start site (Figure S1). The first category of target genes pertains to carbon metabolism, where *aceE* and *acnB* were selected due to their possession of two and one GGA motifs, respectively. The RNA EMSA results demonstrate that CsrA directly binds to *aceE* at 0.0051 nM (400 ng) and to *acnB* at 0.204 nM (1200 ng) (Figure 7A,B). However, their binding affinity was relatively weak as increasing the CsrA concentration did not significantly enhance complex formation, particularly for *acnB*. This weak interaction is likely due to the absence of a CsrA binding motif within its 5′-UTR.

The second category includes genes involved in nitrogen metabolism: *gltB*, *gcvP*, *tdh*, and *thrA* (Figure 7C–F). Among these, CsrA exhibited high-affinity binding to *gltB* and moderate binding to *gcvP* and *tdh*. However, *thrA* did not interact with CsrA despite containing two to three GGA motifs in its sequence. This failure of binding may be attributed to the spatial arrangement of the motifs, which might not conform to the optimal CsrA binding site requirements.

The third category comprises genes associated with bacterial secretion systems, including *tssL*, *tagH*, *cesT*, *hopJ*, *BAU10_07795*, *yopD*, and *sctC* (Figure 7G–L). The EMSA results revealed that *tagH* and *tssL* mRNAs exhibited strong binding to CsrA, suggesting that CsrA negatively regulates the type VI secretion system (T6SS) in *V. alginolyticus* at the post-transcriptional level. Additionally, *cesT*, *hopJ*, *BAU10_07795*, *yopD*, and *sctC* were identified as direct CsrA targets, indicating potential regulation of the type III secretion system (T3SS) through direct mRNA binding, which may either repress or enhance their translation.

Finally, we investigated the cold shock protein *cspD*, which shows dramatic changes upon *csrA* mutation. The EMSA results confirm that CsrA binds to *cspD* with high affinity (Figure 7M), suggesting direct regulation of *cspD* expression by CsrA.

The RNA EMSA analysis identified multiple CsrA target mRNAs across diverse functional pathways, including carbon and nitrogen metabolism, secretion systems, and stress response.

## 4. Discussion

CsrA is a widely conserved RNA-binding protein that acts as a global post-transcriptional regulator in diverse bacterial species. By modulating mRNA stability and translation initiation [5], CsrA enables bacteria to rapidly adjust gene expression in response to environmental and physiological cues. In this study, our integrative transcriptomic and proteomic analyses reveal that CsrA regulates a broad spectrum of genes and pathways in *V. alginolyticus*, encompassing not only central metabolism and virulence factors but also key transcriptional regulators. These findings reinforce the notion that post-transcriptional regulation represents a critical and often underappreciated layer of bacterial gene control, operating in coordination with transcriptional networks to orchestrate complex adaptive responses.

In this study, we identified the CsrA regulon using integrative transcriptomic and proteomic analyses. By comparing gene expression profiles between a *csrA* mutant and its wildtype strain during the early exponential growth phase, we found that CsrA regulates approximately 14% of the *V. alginolyticus* transcriptome and 22% of its proteome. However, the actual number of CsrA-regulated genes may be even higher as the ZJ-T-*csrA*R6H mutant produces a partially functional CsrA protein, which was also manifested in a recent study on *V. cholerae* [26]. Previous studies have suggested that CsrA primarily functions as a repressor during the stationary phase and has limited effects on gene expression during early growth. In *V. cholerae*, a transcriptomic analysis revealed that only 147 genes are differentially expressed in the *csrA* mutant strain during the exponential growth phase, whereas up to 712 DEGs are significantly regulated in the stationary phase [26]. This discrepancy with our findings may stem from species-specific differences in the *V. alginolyticus* Csr system or variations in experimental conditions, particularly differences in growth media (*V. alginolyticus*: LBS vs. *V. cholerae*: M63 + NRES).

Given that CsrA was initially identified for its role in carbon storage regulation in *E. coli* [6], it is not surprising that central carbon metabolism is a key target pathway of CsrA regulation. In *E. coli*, CsrA activates glycolysis at both the mid-exponential phase and the stationary phase by positively regulating the expression and protein production of *pfkA*, which encodes the glycolytic enzyme phosphofructokinase I [7,17,53,54,55]. However, unlike in *E. coli*, we did not observe significant changes in *pfkA* expression in *V. alginolyticus*, possibly due to differences in the bacterial growth phase, as our study focused on early exponential phase cells. In *V. alginolyticus*, key genes involved in gluconeogenesis and glycolysis, including *pdhA* (encoding pyruvate dehydrogenase E1), *acsA* (encoding acetyl-CoA synthase), and *fbp* (encoding fructose-1,6-bisphosphatase I), were significantly upregulated at the transcriptional level. This is consistent with findings in enteropathogenic *E. coli* (EPEC), where a lack of *csrA* led to an accumulation of fructose-6-phosphate (F6P) and increased flux through the glycogen synthesis pathway [55]. At the protein level, PDHA, GAPDH (glyceraldehyde-3-phosphate dehydrogenase), PDHB, and FBP were also upregulated, while most other genes within these pathways remained unaffected by the *csrA* mutation. Additionally, we found that *V. alginolyticus* CsrA represses TCA cycle gene expression during the early exponential phase, a pattern consistent with previous observations in both *E. coli* and *V. cholerae* [17,53]. These findings highlight the conserved yet species-specific regulatory role of CsrA in central carbon metabolism.

While the role of CsrA in central carbon metabolism has been extensively studied, its regulatory influence on amino acid metabolism is often overlooked. The mutation or deletion of *csrA* has been shown to impair the utilization of both carbon and amino acid sources, leading to metabolic deficiencies [27,56,57]. Metabolomic and transcriptomic studies have revealed that pathways linked to the citric acid cycle, including aromatic amino acid biosynthesis, are negatively affected in *csrA* mutants [55]. Additionally, in *V. cholerae*, CsrA plays a key role in regulating amino acid transport and metabolism [26]. Despite these insights, the global regulatory network linking CsrA to amino acid metabolism remains poorly understood.

Our analysis provides compelling evidence that CsrA’s regulatory functions extend beyond the citric acid cycle to influence amino acid biosynthesis, affecting nearly all essential amino acids (16 out of 20), as summarized in Figure 4. The EMSA confirmed that CsrA directly interacts with the mRNAs of *acnB*, *aceE*, *gltB*, *tdh*, and *gcvP*, suggesting a direct post-transcriptional regulatory mechanism. Furthermore, proteomic data provide deeper insights into CsrA’s role in amino acid metabolism, revealing significant changes in protein abundance, even when transcriptional differences were minimal (Figure 4). These findings highlight the broad and intricate influence of CsrA on amino acid metabolism and underscore its role as a global post-transcriptional regulator in *V. alginolyticus*.

Beyond its role in metabolic regulation, *V. alginolyticus* CsrA is a critical regulator of virulence. The type III secretion system (T3SS), a major virulence determinant, facilitates the injection of effector proteins into host cells, inducing apoptosis and oncosis. Mutants lacking a functional T3SS exhibit significantly reduced virulence [58,59]. In our study, eight T3SS-related proteins displayed markedly reduced expression in the *csrA* mutant, suggesting that CsrA positively regulates T3SS activity during the early exponential phase. In contrast, the type VI secretion system (T6SS) serves as a molecular weapon for interbacterial competition, delivering toxic effectors into neighboring cells to confer a competitive advantage [60]. Interestingly, we observed that many T6SS genes were upregulated in the *csrA* mutant, indicating that CsrA may act as a repressor of T6SS during early growth. This regulatory balance suggests that CsrA fine-tunes virulence strategies, promoting host colonization via T3SS while simultaneously modulating interbacterial interactions through T6SS repression.

The CsrA-mediated regulation of secretion systems is a conserved mechanism across diverse bacterial species. For instance, CsrA influences the expression of *cesT*, a T3SS chaperone in *E. coli* [31], and *hrpG*, a master regulator of *hrp/hrc* genes in *Xanthomonas* [44]. In this study, we significantly expanded the known repertoire of CsrA-regulated targets, identifying direct binding interactions with *tagH*, *tssL*, *cesT*, *hopJ*, *BAU10_07795*, *yopD*, and *sctC*. These findings broaden our understanding of CsrA’s regulatory network and its role in coordinating virulence strategies in *V. alginolyticus*.

A particularly striking observation in our study is the extensive alteration in transcription factors in the *csrA* mutant, which plausibly explains how CsrA, despite being a post-transcriptional regulator, exerts broad effects on gene transcription in *V. alginolyticus*. Recent studies have demonstrated that CsrA directly binds to multiple mRNAs encoding transcriptional regulators, including the alternative sigma factors RpoS and RpoE, both of which control the expression of a vast number of genes [16,26]. In our analysis, we observed a significant upregulation of *rpoS*, *rpoE*, *crl*, *dmlR*, *csgD*, *rbcR*, and *lrp* in the csrA mutant. Notably, *lrp* (leucine-responsive regulatory protein), a known direct target of CsrA [17], exhibited a 5.3-fold increase in expression. As a global transcriptional regulator, Lrp senses environmental nutrient availability, primarily amino acids, and modulates the expression of genes involved in metabolism, virulence, motility, nutrient transport, stress tolerance, and antibiotic resistance [61,62]. Previous studies in *E. coli* have shown that Lrp directly activates or represses approximately 10% of genes, with indirect regulatory effects extending to as much as 32% of the genome [61].

In particular, *rraA* (*regulator of ribonuclease activity A*), a known RNase E binding protein, was significantly upregulated in the *csrA* mutant, suggesting a potential link between CsrA and RNase E activity through RraA regulation. Previous studies have shown that *csrA* impairment alters the stability of numerous mRNA candidates in *V. alginolyticus* [27], a phenomenon also reported in other bacteria. This suggests that mRNA degradation may be a key mechanism by which CsrA exerts its regulatory functions. However, the precise mechanism by which CsrA influences mRNA stability remains unclear. In most cases, CsrA binding recruits RNase E and its associated degradosome, leading to the rapid degradation of target mRNAs. RraA (Regulator of ribonuclease activity A), a known RNase E binding protein. In this study, we found that CsrA directly binds the *rraA* 5′-UTR and negatively regulates the expression of *rraA*. These results indicate that CsrA directly binds the *rraA* 5′-UTR, likely promoting mRNA degradation and inhibiting translation initiation. This study reveals a novel regulatory mechanism by which CsrA modulates mRNA stability, enhancing RNase E activity by repressing *rraA* expression, thereby accelerating mRNA degradation in *V. alginolyticus*.

Additionally, we identified a dramatic increase in the expression and protein production of the cold shock protein CspD, with 9.36-fold and 165.34-fold increases, respectively. Cold shock proteins play vital roles in bacterial stress responses, and transcriptomic studies in B. subtilis have revealed that *cspB* and *cspD* influence the expression of approximately 20% of all genes [50]. Here, we confirmed that *cspD* is a novel direct target of CsrA, with CsrA binding specifically to the 5′ untranslated region of *cspD* mRNA to regulate its expression. Taken together, our findings highlight that CsrA’s extensive influence on gene transcription is largely mediated through its regulation of key transcriptional regulators, including sigma factors, global regulators such as Lrp, and stress response proteins like CspD. This regulatory cascade underscores the central role of CsrA in orchestrating complex physiological responses in *V. alginolyticus*.

Overall, our findings not only expand the known CsrA regulon in *V. alginolyticus* but also emphasize the central role of post-transcriptional regulation in coordinating bacterial physiology and pathogenesis. The ability of CsrA to fine-tune central metabolism, virulence secretion systems, and global transcription regulators highlights its function as a master regulator of bacterial adaptability. Understanding such regulatory hierarchies may uncover new targets for antimicrobial intervention, particularly in pathogenic bacteria that rely on rapid environmental responses for survival and virulence. Future studies exploring the interplay between RNA-binding proteins, small RNAs, and transcriptional regulators will be essential to fully elucidate the post-transcriptional regulatory landscape in bacteria.

## 5. Conclusions

This study highlights the pivotal role of CsrA as a global post-transcriptional regulator in *V. alginolyticus*. Through integrative transcriptomic and proteomic analyses, we demonstrate that CsrA orchestrates key cellular processes, including central carbon and amino acid metabolism, quorum sensing, and secretion systems, regulating a substantial portion of the transcriptome and proteome. The identification of direct interactions between CsrA and numerous regulatory and metabolic mRNAs underscores the breadth and complexity of its regulatory network. Together, these results provide a valuable framework for future investigations into bacterial post-transcriptional regulation and its central role in microbial physiology, adaptability, and pathogenesis.

## Figures and Tables

**Table 1:** Strains and plasmids used in this study.

Strains or Plasmids	Relevant Characteristics	Source
*Vibrio alginolyticus*		
ZJ-T	Ap^r^ (ampicillin-resistant), a translucent/smooth variant of the wild strain ZJT51; isolated from diseased *Epinephelus coioides* off the southern China coast	[28]
ZJ-T-*csrA*R6H	Ap^r^; ZJ-T carrying a point mutation that replaces the arginine residue at amino acid position 6 with a histidine (R6H)	[27]
ZJ-T/over *csrA*-pSCT32	Cm^r^; ZJ-T carrying the CsrA overexpression plasmid pSCT32-over-*csrA*	[27]
*E. coli*		
CsrA-6 × his-pET28b/BL21 (DE3)	Kan^r^; *E. coli* BL21 (DE3) carrying the fusion expression plasmid CsrA-6 × his-pET28b	This study
Plasmids		
pET28b	Kan^r^; an expression plasmid with a pBR322 origin, T7 promoter, and 6 × histag	Xiaoxue Wang
CsrA-6 × his-pET28b	Kan^r^; the expression plasmid pET28b carrying the *csrA* open reading frame fusion	This study

## Data Availability

All RNA sequencing data are deposited in GenBank (wildtype biosample: SAMN29675353, SRA: SRR20124473-SRR20124475; *csrA* mutant strain biosample: SAMN4356113, SRA: SRR30591842-SRR30591844) (accessed on 9 September 2024). All proteome data are deposited in the iProX (Integrated Proteome Resources) database (https://www.iprox.cn/page/home.html, accessed on 19 July 2022), and the accession ID is PXD045577. The other data presented in this study are available in the article.

## Supplementary Materials

The following supporting information can be downloaded at https://www.mdpi.com/article/10.3390/microorganisms13071516/s1, Figure S1: GGA motifs of sequences that range from −100 to +100 of the initiate codons; Table S1. Primers in this study; Table S2. Output statistics of sequencing results of transcriptome; Table S3. Significantly different genes in the *csrA* mutant.
