# Supplementary material for: Integrated Transcriptomic and Proteomic Analyses Reveal CsrA-Mediated Regulation of Virulence and Metabolism in Vibrio alginolyticus
Source: Microorganisms. 2025 Jun 28;13(7):1516. doi: 10.3390/microorganisms13071516 (PMC12298160; doi:10.3390/microorganisms13071516)
Supplement: Supplementary file 1 [file microorganisms-13-01516-s001.zip › microorganisms-3694362-supplementary/Figure S1.pdf]

Figure S1. GGA motifs of sequences that range from −100 to +100 of the initiate codons. Bold letters (depicted in blue) indicate the start codon and bold letters (depicted in red) indicate GGA motifs.

aceE:	GTCTCGACGAGAGCGTTCTCTACGTCGAATTCAGCAGGGTAATGACTCGTAAGAGCGTTACTTAAATAAGT<br>AGATCCAACCAACAGAA**GGA**TAGATCGCCATGTCTGAT**ATG**AAGCATGACGTAGATGCACT**GGA**AACTCAA<br>GAATGGCTACAAGCGCTTGAGTCAGTTGTACGTGAAGAAGGTCTAGAGCGTGCTCAAT
acnB	AGCTGCTTATGTAAAAGCGGGTTTCCTAACAGCAGTAGCAAAAGGTGAAGTTAAATCACCATTAGTGAGTC<br>GTGAAAAAGCGGCTGAACTGCTAGGCACT**ATG**CAAGGTGGTTACAACATCGAACCGCTAGTTGAGCTATT**G**<br>**GA**TGATGAAGCACTGGCAGAGATCGCAGTAAAAGCGCTGTCTCATACGCTGCTGATGT
gltB	ACATAAAGCTATAAAGATGACGCATAGTTAGTCATCGATATCAACGTAAGCAGTGTTGATATATCTGTCG**GG**<br>**A**TGAC**GGA**TAAGCAAAG**GGA**GAATTGCAATGGCTCTTT**ATG**ATCCAAGTCTTGAGAAAGACAACTGT**GGA**T<br>TTGGCTTGATCGCGCATAT**GGA**AGGCGAACAAAGCCACAAGCTGGTACGTACAGCAA
gcvP	AGCCTGTTTAGAGAACAGCTTTTTTTCTAGGTTC**GGA**CGTATAATTTAATTTATCAGTAACGCAAATCCGTTA<br>CCCGAACCAA**GGA**GTAGGTAAA**GGA**CA**ATG**ACTGAATTACTTCAAAGCCTCAGCACACAAAACGAGTTCGT<br>TGCTCGCCACAAT**GGA**CCAAACAAATCTGACCAACAGAAAATGTT**GGA**AGCGATCA
tdh	TAGACCGTGCAATCGACGCGTTCATCCAAGTTGGTAA**GGA**CATGGGTCTTATTTAAGATTCTAGGCCTTAG<br>ACAATAGGGCCTTTAATTAAGGTTACATT**ATG**AAAATTAAAGCATTATCAAAGCTAAAGCCTGAAGAAGGCA<br>TCT**GGA**TGACTGAGGTTGATAAACCAGAAGTTGGCCATAACGACATTCTGATCAAAA
thrA	AATTCACAAAAAAGGCCTGTATCCAACAAGATACAGGCCTTTTTTTATGCTTTTTTATCCAGAATTTAGACAA<br>ATTTAAGAAGATATACC**GGAGGA**AG**GGATG**CGAGTATTGAAGTTTGGC**GGA**TCATCACTAGCTGATGCAG<br>ATCGCTTTTTGAGAGCGGCAGACATTATTGCCAATAACGCTCAACAAGAAGAAGTAG
yopD	GACGTATTCGCCGATGAAGAACAACGACAGAATTTAATCCAAGCGGTACAAAAGGCGCTAGACGAAGCCA<br>TTGATATAGAAGAAGA**GGA**AGCGTG**GGA**AG**ATG**AGTT**GGA**TTGATGCATCCGTCGATGAATTTTGCCGA**G**<br>**GA**ATGGGGCTCGATGCCGTCGATTTCTCATCAGCT**GGA**CGGGTGCAGCT**GGA**TTTTGAGC
07795	GACGTATTCGCCGATGAAGAACAACGACAGAATTTAATCCAAGCGGTACAAAAGGCGCTAGACGAAGCCA<br>TTGATATAGAAGAAGA**GGA**AGCGTG**GGA**AG**ATG**AGTT**GGA**TTGATGCATCCGTCGATGAATTTTGCCGA**G**<br>**GA**ATGGGGCTCGATGCCGTCGATTTCTCATCAGCT**GGA**CGGGTGCAGCT**GGA**TTTTGAGC
cesT	CACAATGAAGGCGAAAAAGTGACAAAAATAAAGCCATTACCGACATCGTTCAATCTGCAATCGTTGTTCAAT<br>AGAGTTATTGAAACAAAA**GGA**AAAAGTT**ATG**AACACGATTCAACAACTGCTCTT**GGA**GTTTTGCCAGCTCAA<br>TGAGTTGCCCGAACTGAATTTTGAAGAAAATGAACGTTGTCAGCTACTGGTCGATG
sctC	CTGCTTTGCTTTCTAAGCACGTCAATCTCTGTGAGCGTTACATTGCATTATT**GGA**ACAAGAACGTGTGAACC<br>ACACAATCAACCATACAGTGTGGTTACC**ATG**ATGCTAAAAATCTTTCCCAACATTAGACGTGCTGCAACAAA<br>GGCATTGTTATGCGCAGTGAGTGTTGTTCCTTTG**GGA**TATGCGCAAGCAACAGACT
tagH	CAATGTCAGTATTAAGATTTTTATTAATCTTACTGTTAATAAGAAAGCAGTTAATAATAAAATTAAAAATAATA<br>TCTAGCAGGTCTTTGGCGAGGTTTAC**ATG**AAGTTGGTTTTAACCATAACAAGTTTTCACAAATTTACTCCAG<br>AGATTGAGAGTGAGTTCGTGTTTGAATCAAGTGATGAACATGCCAAGGTTACAT
tssL	TGAGGCTAAAAGATAGTGGCGGTATTGCGCTTCACCTATCAGGGCATTTTCCGAATGCTGAACTTGAGCTT<br>T**GGA**GTATTAATCAATAGGCGCGCTCTGT**ATGGA**ACAAACGATAGTAAAACCAACACCGGGT**GGA**AGAGC<br>ACCCGCATCGAAACCACAATCAGAAAAGTCTGT**GGA**TAATACCGTAGTTATCTCAAAAA
cspD	TGACGTTTGTTGGCATTCGCTGACAATGGTAGTACCAAGTTAAAGGTATAACTTGGTTGCAGTAACAACATC<br>AGTAAAAAATGCATGAG**GGA**TGTAAAGC**ATG**GCTACAGGTACAGTAAAGTGGTTTAACAATGCCAAA**GGA**T<br>TTGGGTTTATTTGTTCAGATGAAGAAGAG**GGA**GATATCTTTGCCCACTACTCAACTA
