# Supplementary material for: Integrated Transcriptomic and Proteomic Analyses Reveal CsrA-Mediated Regulation of Virulence and Metabolism in Vibrio alginolyticus
Source: Microorganisms. 2025 Jun 28;13(7):1516. doi: 10.3390/microorganisms13071516 (PMC12298160; doi:10.3390/microorganisms13071516)
Supplement: Supplementary file 1 [file microorganisms-13-01516-s001.zip › microorganisms-3694362-supplementary/Table S1.pdf]

Table S1. Primers in this study

Purpose	Primer name	5' to 3'
CsrA recombinant protein construction	pET28b_F	CACCACCACCACCACCAC
	pET28b_R	GGTATATCTCCTTCTTAAAGTTAAACAAAATTATTTC
	CsrA-ORF_fwd	TTTAAGAAGGAGATATACCATGCTAATTTTGACTCGCCGC
	CsrA-ORF_rev	TCAGTGGTGGTGGTGGTGGTGGTAGTTACCTGAAGCAACGTTGC
	pET28b-check-F	AAGTGGCGAGCCCGATCTTC
	pET28b-check-R	CTAGGGCGCTGGCAAGTGTA
qPCR	q-*16S*-F	CTGGAACTGAGACACGGTCC
	q-*16S*-R	CTCGCACCCTCCGTATTACC
	q-*ompV*-F	TGGAACGTAGAAGCTGGTGT
	q-*ompV*-R	TTTTCGCTGTATCACCGTCG
	q-*tssC1*-F	GTTTGGTAGTGCTGGTGGTG
	q-*tssC1*-R	GCCGAATAGTGCTGGTGAAG
	q-*tssB1*-F	CCTGCTGCGATCGTAAATCA
	q-*tssB1*-R	TCCGCTCCTTCAGTACTGTC
	q-*tagH*-F	TTGACCTAGGCCTTAGTGCT
	q-*tagH*-R	CACCCCTCTAACAAAAGCCG
	q-*artP*-F	ACCTATGGCCGCATATGACA
	q-*artP*-R	CAGCGGTCGGTTCATCAAAA
	q-*argT*-F	ATCGAAGGTGCTTACCCTCC
	q-*argT*-R	TACGTGCAAGCAGAGAAGGA
	q-*csgD*-F	TCCGAAGTGCATCAGTGACA
	q-*csgD*-R	TGCCGTACACAAGTAGCTCT
	q-*lrp*-F	CAGTTCCTGGATGCATCACT
	q-*lrp*-R	ACGTATCGCCCAATAGTCGG
	q-*ousV*-F	AGATATCGCCCACATCTCCG
	q-*ousV*-R	CCCACTCTTTCTAAGGCGGA
	q-*oprF*-F	AACGCCAAAATGCAACAACG
	q-*oprF*-R	GCTGAACTTGTACGCCAGAG
	q-*ompA3*-F	CTTGTAGTAACGGGGCATGC
	q-*ompA3*-R	GAGTGGCAGGTTATCTCCGA
	q-*ompA*-F	ACGTTGGTGGTAAGATGGGT
	q-*ompA*-R	TGGCCACCGAAAGAAGTTTG
	q-*ompP1*-F	TGACTCAGATCGCCAATGGT
	q-*ompP1*-R	TTTGAGCGTCACTTGGGTTG
	q-*ompT*-F	CAAGCGAGCGTACAGATGAC
	q-*ompT*-R	ATCGTTGTTCCAAGCGTGAC
	q-*ompN2*-F	GCCAGTCGTTTTCAACTCGT

	q-*ompN2*-R	TTTCAGCACGACCAGTACCT
	q-*ompN1*-F	CCTTGCTGCGGTTTATGGTT
	q-*ompN1*-R	GTGCCATTTCTTCACGAGCA
	q-*ompW*-F	TAGCAGCAACACCATTCAGC
	q-*ompW*-R	TGAGACCTGCACCAACGTAT
	q-*ompA2*-F	TGACTACACTTGACGACGCT
	q-*ompA2*-R	AAAGCCGTTAGATTGCCGTC
	q-*tssL*-F	TAGCAGTCAAGGTGGGGAAG
	q-*tssL*-R	TCAATTTCCGGTGCGATTCC
	q-*tssM1*-F	AGCGGTTCGAAGGATCATCT
	q-*tssM1*-R	TCCACAAACGGTTCGAGGTA
	q-*hcp*-F	GCAACAGCCGAGCAATACAA
	q-*hcp*-R	TCAACCTTCTCACCCTGCTT
	q-*tssA*-F	GACGAAGATAAGCGCCGAAA
	q-*tssA*-R	GCTAGGAGTCACATCACCGA
	q-*tssC2*-F	CGGCCCAATTTGAACAGGAA
	q-*tssC2*-R	TACGGCAGCAAAACAGTACG
	q-*07795*-F	CCGTCGATGAATTTTGCCGA
	q-*07795*-R	TGCCAAGATAACGCTCGAGA
	q-*yopD*-F	CAAGTCACCATCAGATGCGG
	q-*yopD*-R	CCGCTAATACACCCGAAACG
	q-*sctC*-F	CAACCGTTCCGCTACTATGC
	q-*sctC*-R	AGACATGTAAGACGGCACCA
	q-*cesT*-F	AGGAATAGGTAGTGGCAGCC
	q-*cesT*-R	GAGGTGGTTGGAATCGCTTG
	q-*hopJ*-F	AACAATGCAGGCGAGAACAA
	q-*hopJ*-R	CTTCCCACCCTGTCACCATA
	q-*crl*-F	CGACTCACTTTCGCTTGATGT
	q-*crl*-R	AGTAAAGCCTCCCTCGATGG
	q-*dmlR*-F	CAACTTGGCGTAACACTCGT
	q-*dmlR*-R	AGAGGCTATAGAGTTGGGCA
	q-*rpoS*-F	CCACAGAAAGCAAAGCGACT
	q-*rpoS*-R	TGCTAGACCACGGTTACTGT
	q-*yeaG*-F	GGCAGACCTTCCGTAACAAC
	q-*yeaG*-R	TGGACGAGTTTTCAGGCTCT
	q-*rpoE*-F	GCGCAGTGATTGGAACGATT
	q-*rpoE*-R	CAAACTGTCGCCATGGTCTC
	q-*rsd*-F	GGGTGGCTCAAGTGATGTCA
	q-*rsd*-R	ATCAACGAGGTGCTGACAGA
	q-*hlyU*-F	GCATAACCAAGAGCTGTCCG
	q-*hlyU*-R	ACGCTTTCACTTCATCACTCT
	q-*nhaR*-F	AACGCCTTTCTCAGCACAAG
	q-*nhaR*-R	GCTTGAAGGCCTTGACGATC

	q-*dctD*-F	GACGCGTTGATCTTGTTCGA
	q-*dctD*-R	CTTCGGTTGTTGAGGATGGC
	q-*cspD*-F	TGGTTTAACAATGCCAAAGGATT
	q-*cspD*-R	TACTCGCGTGACAGCCTTT
	q-*proX*-F	TTTTGGGTTCCCGCCTATCT
	q-*proX*-R	GGAGGTGATTTGGCATGTCC
	q-*aotP*-F	TTTGACGAGCCAACTTCTGC
	q-*aotP*-R	CGTTCTGAATCCGGGTTTGT
	q-*braF*-F	ATCTTCGCTGGAGATGACGT
	q-*braF*-R	TTCCACATATTTGCGGTGCG
	q-*putP*-F	TCAGACATGAGTGGTTGGCT
	q-*putP*-R	GTTAGCGCGTCAGTTTGGAT
	q-*artI1*-F	TGTATTTGGCGGCTCAATCG
	q-*artI1*-R	GCTGGCGATGGTTTTATGCT
	q-*artI2*-F	CGTACGCCCCATTCGAATAC
	q-*artI2*-R	AAACGCGCCTCAGTAATGTC
	q-*ousX*-F	GTGGCGTTTTAGTTCCAGGG
	q-*ousX*-R	AGCAGCAGGGTTGTTTTCTG
	q-*oppF*-F	TCAGCATTGCCTCAAAGTCG
	q-*oppF*-R	CTACTTGGCCGTCAGTAGCT
	q-*oppD*-F	TTGCAGGTTCGTGTGACAAG
	q-*oppC*-R	GTCGGCAGAATTTCACCCTC
	q-*oppA*-F	AAAGCCTGCGGAGAAACAAG
	q-*oppA*-R	TGCAACACCAGGGATAGTGT
	q-*oppC*-F	TTATCGAAGCGGCACATGTG
	q-*oppC*-R	GCTGCCAGATTGCTACTTCC
	q-*dppC*-F	TGGATTCTGAGTTGCCACCT
	q-*dppC*-R	ACTGAAGACCGACGGCTAAA
	q-*gsiA*-F	GTGTTGTTGGCGAATCAGGT
	q-*gsiA*-R	AAGTGGATTGAGCGAGGTCA
	q-*yejF*-F	CAACCTCTGCATTAGACCGC
	q-*yejF*-R	GCCGTACCTTGTTCCATGAC
	q-*dppB*-F	GTGATCAGTTTGGTGGCGTT
	q-*dppB*-R	CATTACCCACAAAGCGCGTA
	q-*oppF*-F	ACCTACACATGGCGAGTTGA
	q-*oppF*-R	CACGCTTTTCTACCTCTGCC
	q-*mppA*-F	AAACTGGTTCCTCGCTACGA
	q-*mppA*-R	GTCGAACTCAGGCCAGTACT
	q-*fiu*-F	CCGCATCAATTCCGACACAT
	q-*fiu*-R	ACAGTGCCGCATTCAACATT
	q-*hgbA*-F	GTTTGGTGCGCGTGTTTATG
	q-*hgbA*-R	CATACCCCAGCTTAGCCAGA
	q-*fecA*-F	CTTCTGCCACATCCATGAGC

	q-*fecA*-R	GTTATCAATCGAGCCTGCGG
	q-*fatB*-F	GGCGTTGAGTCTCGTTATGG
	q-*fatB*-R	CCGCATCAATGACAGGCTTT
	q-*feoA*-F	CATGGTAATGGGGCTGCTTC
	q-*feoA*-R	TATTGCATGACACTCGCTCC
	q-*viuB*-F	CCAAAGGCTACGCTGTGATC
	q-*viuB*-R	CATTGCGGAAATATTGGCGC
**RNA-EMSA primers**		
aceE EMSA oligo	T7-*aceE*-F	GATCACTAATACGACTCACTATAGGGGTCTCGACGAGAGCGT
	T7-*aceE*-R	ATTGAGCACGCTCTAGACCTTC
acnB EMSA oligo	T7-*acnB*-F	GATCACTAATACGACTCACTATAGGGAGCTGCTTATGTAAAAGCG
	T7-*acnB*-R	ACATCAGCAGCGTATGAGACAG
gltB EMSA oligo	T7-*gltB*-F	TAATACGACTCACTATAGGGACATAAAGCTATAAAGATGA
	T7-*gltB*-R	TTGCTGTACGTACCAGCTTGT
gcvP EMSA oligo	T7-*gcvP*-F	GATCACTAATACGACTCACTATAGGGAGCCTGTTTAGAGAACAGC
	T7-*gcvP*-R	TGATCGCTTCCAACATTTTCTG
tdh EMSA oligo	T7-*tdh*-F	GATCACTAATACGACTCACTATAGGGTAGACCGTGCAATCGACGCG
	T7-*tdh*-R	TTTTGATCAGAATGTCGTTATGGCC
thrA EMSA oligo	T7-*thrA* -F	GATCACTAATACGACTCACTATAGGGAATTCACAAAAAAGGCCTG
	T7-*thrA*-R	CTACTTCTTCTTGTTGAGCGT
tssL EMSA oligo	T7-*tssL*-F	TAATACGACTCACTATAGGGTGAGGCTAAAAGATAGTGGC
	T7-*tssL*-R	TTTTTGAGATAACTACGGTATTATCC
tagH EMSA oligo	T7-*tagH*-F	TAATACGACTCACTATAGGGCAATGTCAGTATTAAGATTT
	T7-*tagH*-R	ATGTAACCTTGGCATGTTCATC
cesT EMSA oligo	T7-*cesT*-F	TAATACGACTCACTATAGGGCACAATGAAGGCGAAAAAGTG
	T7-*cesT*-R	CATCGACCAGTAGCTGACAA
07795 EMSA oligo	T7-*07795*-F	TAATACGACTCACTATAGGGGACGTATTCGCCGATGAAGA
	T7-*07795*-R	GCTCAAAATCCAGCTGCACCC
yopD EMSA oligo	T7-*yopD*-F	TAATACGACTCACTATAGGGTTCCAAGAGTTGATGAGCCT
	T7-*yopD*-R	CAAGCTTTGTTTCCGGCGCTT
sctC EMSA oligo	T7-*sctC*-F	TAATACGACTCACTATAGGGCTGCTTTGCTTTCTAAGCAC

	T7-*sctC*-R	AGTCTGTTGCTTGCGCATATC
cspD EMSA oligo	T7-*cspD*-F	TAATACGACTCACTATAGGGTGACGTTTGTTGGCATTCGCT
	T7-*cspD*-R	TAGTTGAGTAGTGGGCAAAGA
