# Supplementary material for: Integrated Transcriptomic and Proteomic Analyses Reveal CsrA-Mediated Regulation of Virulence and Metabolism in Vibrio alginolyticus
Source: Microorganisms. 2025 Jun 28;13(7):1516. doi: 10.3390/microorganisms13071516 (PMC12298160; doi:10.3390/microorganisms13071516)
Supplement: Supplementary file 1 [file microorganisms-13-01516-s001.zip › microorganisms-3694362-supplementary/Table S2.pdf]

Table S2. Output statistics of sequencing results of transcriptome.

Feature	ZJ-T	ZJ-T-*csrA*R6H
Raw Reads	21,616,469	22,335,588
Clean Reads	21,407,583	22,104,111
Q20 (%)	98.43%	98.41%
Q30 (%)	95.03%	94.97%
GC content (%)	46.79%	46.73%
Reads mapped to genome (%)	95.99%	96.01%
