# Supplementary material for: Integrated Transcriptomic and Proteomic Analyses Reveal CsrA-Mediated Regulation of Virulence and Metabolism in Vibrio alginolyticus
Source: Microorganisms. 2025 Jun 28;13(7):1516. doi: 10.3390/microorganisms13071516 (PMC12298160; doi:10.3390/microorganisms13071516)
Supplement: Supplementary file 1 [file microorganisms-13-01516-s001.zip › microorganisms-3694362-supplementary/Table S3.pdf]

Table S3 Significantly differential genes in *csrA* mutant

Locus tag	Symbol	Gene product	P-FC	FDR	TR-FC	FDR
**<u>Metabolism</u>**						
**carbohydrate metabolism**						
BAU10_19305	*glgC2*	glucose-1-phosphate adenylyltransferase	8.74	0.0300	2.21	0.0000
BAU10_23995	*ygfF*	glucose 1-dehydrogenase	6.45	0.0059	/	/
BAU10_13680	*ppc*	phosphoenolpyruvate carboxylase	5.70	0.0000	/	/
BAU10_01550	*tas*	oxidoreductase Tas, aldo/keto reductase family protein	4.05	0.0000	/	/
BAU10_23955	*glxK*	glycerate kinase	2.72	0.0011	3.53	0.0000
BAU10_04115	*glgC1*	glucose-1-phosphate adenylyltransferase	2.39	0.0000	/	/
BAU10_00575	*fbp*	Fructose-1,6-bisphosphatase	1.98	0.0000	2.00	0.0000
BAU10_22910	*malS*	alpha-amylase	1.95	0.0000	/	/
BAU10_17690	*gap2*	glyceraldehyde-3-phosphate dehydrogenase	1.84	0.0000	/	/
BAU10_04120	*glgA*	starch synthase	1.77	0.0001	/	/
BAU10_11020	*glpK*	glycerol kinase	1.72	0.0000	3.47	0.0000
BAU10_00850	*mtlD*	mannitol-1-phosphate 5-dehydrogenase	1.65	0.0482	/	/
BAU10_22985	*pulA*	pullulanase-type alpha-1,6-glucosidase	1.64	0.0000	/	/
BAU10_03720	*lplT*	acyl-phosphate glycerol 3-phosphate acyltransferase	1.62	0.0147	/	/
BAU10_07700	*prpB*	carboxyphosphonoenolpyruvate phosphonomutase	/	/	3.85	0.0000
BAU10_07685	*prpF*	2-methylaconitate cis-trans isomerase PrpF	/	/	2.35	0.0000
BAU10_19235	*fruK*	1-phosphofructokinase	/	/	2.87	0.0043
BAU10_04715	*oadB*	oxaloacetate decarboxylase, beta subunit	/	/	-3.76	0.0000
BAU10_12260	*oadB*	oxaloacetate decarboxylase, beta subunit	/	/	3.29	0.0000
BAU10_19230	*fruA*	PTS fructose transporter subunit IIBC	/	/	4.70	0.0000
BAU10_15030	--	Acetolactate synthase small subunit	/	/	2.18	0.0001
**other carbon source utilization**						
BAU10_12270	*oadG*	putative oxaloacetate decarboxylase subunit gamma	97.34	0.0001	/	/
BAU10_17540	--	1,4-alpha-glucan branching protein	8.48	0.0005	4.46	0.0000

Locus tag	Symbol	Gene product	P-FC	FDR	TR-FC	FDR
BAU10_21170	--	putative acetyltransferase	5.85	0.0000	3.37	0.0000
BAU10_07695	*prpC*	1 2-methylcitrate synthase	5.18	0.0004	2.79	0.0000
BAU10_05815	*cobS*	adenosylcobinamide-GDP ribazoletransferase	4.64	0.0179	/	/
BAU10_13815	*yheT*	hydrolase	3.37	0.0000	/	/
BAU10_21000	*slr1608*	PQQ-dependent sugar dehydrogenase	3.31	0.0000	/	/
BAU10_20990	*elbB*	isoprenoid biosynthesis glyoxalase ElbB	3.05	0.0000	/	/
BAU10_20365	*yghX*	dienelactone hydrolase family protein	2.68	0.0126	5.37	0.0000
BAU10_07680	*PA3568*	propionyl-CoA synthetase	2.51	0.0000	2.17	0.0000
BAU10_20575	*gucD*	aldehyde dehydrogenase family protein	2.50	0.0233	6.01	0.0000
BAU10_05905	*aldH*	aldehyde dehydrogenase (NADP(+))	2.46	0.0009	4.80	0.0000
BAU10_07690	*acnM*	Fe/S-dependent 2-methylisocitrate dehydratase AcnD	2.37	0.0000	2.58	0.0000
BAU10_22325	*lldD*	L-lactate dehydrogenase (cytochrome)	2.36	0.0000	2.13	0.0000
BAU10_06000	*VC_1345*	homogentisate 1,2-dioxygenase	2.26	0.0000	/	/
BAU10_05810	*cobT*	nicotinate-nucleotide--dimethylbenzimidazole phosphoribosyltransferase	2.20	0.0130	/	/
BAU10_03435	*mltB*	Membrane-bound lytic murein transglycosylase B	2.15	0.0018	/	/
BAU10_06005	--	fumarylacetoacetate hydrolase family protein	1.90	0.0009	/	/
BAU10_05820	*cobP*	bifunctional adenosylcobinamide kinase/adenosylcobinamide-phosphate guanylyltransferase	1.78	0.0022	/	/
BAU10_20775	*ycjY*	dienelactone hydrolase	1.74	0.0003	/	/
BAU10_11995	*chbP*	N,N'-diacetylchitobiose phosphorylase	1.67	0.0206	/	/
BAU10_18970	*calB*	coniferyl aldehyde dehydrogenase	1.63	0.0000	/	/
BAU10_10010	*pdxB*	4-phosphoerythronate dehydrogenase	1.55	0.0000	/	/
BAU10_21550	*otnI*	hydroxypyruvate isomerase family protein	1.54	0.0000	/	/
BAU10_13490	*cysH*	phosphoadenosine phosphosulfate reductase	-4.57	0.0000	/	/
BAU10_04725	*oadG2*	oxaloacetate decarboxylase	-4.29	0.0238	-2.99	0.0000
BAU10_09905	*gloA*	putative lactoylglutathione lyase	-3.60	0.0001	/	/
BAU10_13495	*cysI*	assimilatory sulfite reductase (NADPH) hemoprotein subunit	-3.58	0.0000	/	/

Locus tag	Symbol	Gene product	P-FC	FDR	TR-FC	FDR
BAU10_09555	*ackA1*	acetate/propionate kinase	-2.42	0.0000	/	/
BAU10_24010	*torZ*	molybdopterin guanine dinucleotide-containing S/N-oxide reductase	-2.41	0.0001	/	/
BAU10_24320	*ldhA*	2-hydroxyacid dehydrogenase	-1.91	0.0000	-2.15	0.0000
BAU10_19015	*gloA*	lactoylglutathione lyase	-1.90	0.0080	/	/
BAU10_04980	*bioD*	dithiobiotin synthetase	-1.85	0.0000	/	/
BAU10_19010	*nemA*	alkene reductase	-1.74	0.0158	/	/
BAU10_22340	*cah*	carbonic anhydrase	-1.73	0.0000	/	/
BAU10_09560	*pta*	phosphate acetyltransferase	-1.69	0.0004	/	/
BAU10_10535	*mltD*	Membrane-bound lytic murein transglycosylase D	-1.55	0.0364	/	/
BAU10_17815	--	Poly(3-hydroxyalkanoate) polymerase	/	/	3.94	0.0000
**central&energy metabolism**						
BAU10_13825	*prkB*	phosphoribulokinase	9.75	0.0000	/	/
BAU10_05640	*ydiJ*	FAD-binding oxidoreductase	7.97	0.0000	/	/
BAU10_04060	*icd2*	NADP-dependent isocitrate dehydrogenase	5.88	0.0000	2.69	0.0000
BAU10_22450	--	2-oxoglutarate dehydrogenase complex, dehydrogenase component	5.83	0.0095	/	/
BAU10_00570	*ppa*	inorganic pyrophosphatase	5.57	0.0000	3.33	0.0000
BAU10_03265	*gltA*	type II citrate synthase	5.29	0.0000	6.65	0.0000
BAU10_03305	*sucD*	succinyl-CoA synthetase alpha subunit	3.93	0.0000	3.03	0.0000
BAU10_12035	*acnB*	bifunctional aconitate hydratase 2/2-methylisocitrate dehydratase	3.81	0.0000	3.54	0.0000
BAU10_20475	*pdhB*	putative pyruvate dehydrogenase E1 component, beta subunit	3.79	0.0001	/	/
BAU10_03300	*sucC*	succinyl-CoA synthetase subunit beta	3.70	0.0000	3.38	0.0000
BAU10_20485	*etfA*	electron transfer flavoprotein subunit alpha	3.62	0.0000	3.48	0.0000
BAU10_03275	*sdhD*	succinate dehydrogenase, hydrophobic membrane anchor protein	3.44	0.0129	5.80	0.0000
BAU10_03280	*sdhA*	succinate dehydrogenase catalytic subunit	3.38	0.0000	5.81	0.0000
BAU10_03285	*sdhB*	succinate dehydrogenase catalytic subunit	3.34	0.0000	5.99	0.0000
BAU10_20490	*PA2953*	electron transfer flavoprotein-ubiquinone oxidoreductase	2.43	0.0000	4.94	0.0000
BAU10_20470	*pdhA*	pyruvate dehydrogenase (acetyl-transferring) E1 component subunit alpha	2.40	0.0000	4.61	0.0011

Locus tag	Symbol	Gene product	P-FC	FDR	TR-FC	FDR
BAU10_03290	*sucA*	2-oxoglutarate dehydrogenase, E1 component	2.39	0.0000	2.53	0.0000
BAU10_03295	*sucB*	dihydrolipoamide acetyltransferase	2.35	0.0000	2.18	0.0000
BAU10_18015	*nnr*	bifunctional ADP-dependent NAD(P)H-hydrate dehydratase/NAD(P)H-hydrate epimerase	2.25	0.0045	/	/
BAU10_19755	*pntA*	Re/Si-specific NAD(P)(+) transhydrogenase subunit alpha	1.97	0.0000	/	/
BAU10_00635	*mdh*	malate dehydrogenase	1.96	0.0000	2.77	0.0000
BAU10_05000	--	NAD(P)/FAD-dependent oxidoreductase	1.84	0.0206	/	/
BAU10_20610	*cyoB*	cytochrome o ubiquinol oxidase subunit I	1.76	0.0000	/	/
BAU10_06620	*ydgJ*	oxidoreductase	1.75	0.0210	/	/
BAU10_20615	*cyoA*	cytochrome o ubiquinol oxidase, subunit II	1.65	0.0063	/	/
BAU10_09745	--	SDR family oxidoreductase	1.65	0.0000	/	/
BAU10_21745	*nadE*	ammonia-dependent NAD(+) synthetase	1.51	0.0000	/	/
BAU10_04720	*oadA1*	oxaloacetate decarboxylase alpha subunit	-6.12	0.0000	-3.34	0.0000
BAU10_13500	*cysJ*	sulfite reductase [NADPH] flavoprotein, alpha-component	-3.66	0.0000	/	/
BAU10_14120	*frdD*	fumarate reductase subunit D	-2.82	0.0002	/	/
BAU10_14110	*frdB*	succinate dehydrogenase	-2.72	0.0206	-2.38	0.0000
BAU10_14105	*frdA*	fumarate reductase	-2.70	0.0000	-2.47	0.0000
BAU10_18515	*yfkO*	NAD(P)H-dependent oxidoreductase	-1.81	0.0087	/	/
BAU10_13710	*maeB*	putative malate oxidoreductase	-1.68	0.0002	-2.09	0.0000
BAU10_20590	*Dred_2421*	NADH:flavin oxidoreductase	-1.58	0.0216	/	/
BAU10_17840	*napA*	periplasmic nitrate reductase	/	/	3.52	0.0000
BAU10_17835	*napB*	periplasmic nitrate reductase, cytochrome c-type protein	/	/	3.12	0.0000
BAU10_20015	*nirD*	nitrite reductase [NAD(P)H], small subunit	/	/	2.97	0.0001
BAU10_21565	*ltnD*	NAD(P)-dependent oxidoreductase	/	/	-2.02	0.0003
BAU10_06785	*HVO_B0029*	Gfo/Idh/MocA family oxidoreductase	/	/	-2.40	0.0358
BAU10_20020	*nirB*	nitrite reductase large subunit	/	/	2.05	0.0003

Locus tag	Symbol	Gene product	P-FC	FDR	TR-FC	FDR
BAU10_03270	*sdhC*	succinate dehydrogenase cytochrome b556 large membrane subunit	/	/	5.39	0.0000
BAU10_24005	*torY*	cytochrome c	/	/	-2.01	0.0000
BAU10_18305	*etfB*	electron transfer flavoprotein, beta-subunit	/	/	3.24	0.0018
BAU10_14275	*acsA*	acetate--CoA ligase	/	/	13.98	0.0000
**Amino acid metabolism**						
BAU10_01395	*gltB*	glutamate synthase large subunit	140.92	0.0000	13.40	0.0000
BAU10_01390	*gltD*	glutamate synthase subunit beta	76.53	0.0000	12.90	0.0000
BAU10_18860	--	putative acetyltransferase	19.78	0.0000	7.40	0.0000
BAU10_01385	*gltB*	glutamate synthase large subunit	10.84	0.0000	/	/
BAU10_01380	*gltD*	glutamate synthase	10.07	0.0000	2.01	0.0000
BAU10_13845	*astA*	putative arginine/ornithine succinyltransferase	9.68	0.0000	10.59	0.0000
BAU10_18550	*gdhA*	NADP-specific glutamate dehydrogenase	9.27	0.0000	9.52	0.0000
BAU10_13840	*astD*	succinylglutamate 5-semialdehyde dehydrogenase	7.22	0.0000	13.25	0.0000
BAU10_05560	*csd*	cysteine desulfurase-like protein	6.86	0.0000	5.44	0.0000
BAU10_03770	*cysM*	cysteine synthase/cystathionine beta-synthase family protein	6.77	0.0026	3.68	0.0000
BAU10_13120	*arcA*	arginine deiminase	6.07	0.0000	/	/
BAU10_13850	*argD*	bifunctional N-succinyldiaminopimelate-aminotransferase/acetylornithine transaminase protein	5.33	0.0000	5.49	0.0000
BAU10_12545	*serA*	phosphoglycerate dehydrogenase	5.32	0.0000	2.50	0.0000
BAU10_06145	*puuE*	4-aminobutyrate--2-oxoglutarate transaminase	5.24	0.0039	2.90	0.0000
BAU10_13440	*lysC*	lysine-sensitive aspartokinase 3	4.93	0.0000	/	/
BAU10_00740	*leuA*	2-isopropylmalate synthase	4.90	0.0000	/	/
BAU10_00770	*ilvI*	acetolactate synthase, large subunit, biosynthetic type	4.83	0.0000	3.75	0.0000
BAU10_23400	--	1-pyrroline-5-carboxylate dehydrogenase	4.57	0.0000	2.23	0.0000
BAU10_11125	*VC_2345*	phosphoserine phosphatase	4.49	0.0001	/	/
BAU10_10385	*dapE*	succinyl-diaminopimelate desuccinylase	4.38	0.0000	/	/
BAU10_17260	*doeD*	aspartate aminotransferase family protein	4.22	0.0000	/	/

Locus tag	Symbol	Gene product	P-FC	FDR	TR-FC	FDR
BAU10_20645	*mmsA*	CoA-acylating methylmalonate-semialdehyde dehydrogenase	4.21	0.0000	2.93	0.0000
BAU10_08665	*aspC*	aspartate aminotransferase	4.18	0.0000	/	/
BAU10_02090	*met17*	O-acetylhomoserine aminocarboxypropyltransferase/cysteine synthase	4.10	0.0000	4.33	0.0000
BAU10_23405	*putA*	bifunctional proline dehydrogenase/L-glutamate gamma-semialdehyde dehydrogenase PutA	4.05	0.0000	/	/
BAU10_00775	*ilvH*	acetolactate synthase small subunit	3.99	0.0029	3.95	0.0000
BAU10_15035	*ilvE*	branched-chain amino acid aminotransferase	3.90	0.0000	2.06	0.0000
BAU10_09765	*asd1*	aspartate-semialdehyde dehydrogenase	3.68	0.0000	/	/
BAU10_05910	*VP1330*	4-hydroxyproline epimerase	3.64	0.0019	5.87	0.0000
BAU10_05285	*metC*	cystathionine beta-lyase	3.24	0.0000	/	/
BAU10_20960	*phhA*	phenylalanine 4-monooxygenase	3.13	0.0029	/	/
BAU10_20625	*mmsB*	3-hydroxyisobutyrate dehydrogenase	3.11	0.0000	3.48	0.0000
BAU10_18345	*paaK*	putative phenylacetate-CoA ligase	3.06	0.0000	/	/
BAU10_06210	*metAS*	homoserine O-succinyltransferase	3.05	0.0011	/	/
BAU10_15045	*ilvA*	threonine ammonia-lyase, biosynthetic	2.98	0.0000	2.12	0.0000
BAU10_13445	*metH*	methionine synthase	2.93	0.0000	/	/
BAU10_00735	*leuB*	3-isopropylmalate dehydrogenase	2.92	0.0000	/	/
BAU10_14085	--	acetyltransferase	2.83	0.0000	/	/
BAU10_08960	*trpE*	anthranilate synthase component 1	2.83	0.0001	/	/
BAU10_13595	*aroB*	3-dehydroquinate synthase	2.77	0.0000	/	/
BAU10_01450	*thrC*	threonine synthase	2.71	0.0000	/	/
BAU10_15320	*ilvC*	ketol-acid reductoisomerase	2.69	0.0000	2.35	0.0000
BAU10_05530	*serC*	phosphoserine aminotransferase	2.68	0.0001	/	/
BAU10_06530	*ectB*	Diaminobutyrate--2-oxoglutarate transaminase	2.66	0.0003	2.33	0.0000
BAU10_19820	*tyrB*	aspartate/tyrosine/aromatic aminotransferase	2.56	0.0000	/	/
BAU10_01695	*tyrA*	bifunctional chorismate mutase/prephenate dehydrogenase	2.56	0.0007	/	/
BAU10_15040	*ilvD*	dihydroxy-acid dehydratase	2.51	0.0000	/	/

Locus tag	Symbol	Gene product	P-FC	FDR	TR-FC	FDR
BAU10_05085	*hisB*	bifunctional histidinol-phosphatase/imidazoleglycerol-phosphate dehydratase HisB	2.44	0.0001	/	/
BAU10_05070	*hisG*	ATP phosphoribosyltransferase	2.41	0.0000	/	/
BAU10_15025	*ilvG*	acetolactate synthase II large subunit	2.38	0.0000	/	/
BAU10_05100	*hisF1*	imidazole glycerol phosphate synthase subunit HisF	2.32	0.0000	/	/
BAU10_13700	*metB*	cystathionine gamma-synthase	2.27	0.0053	/	/
BAU10_13695	*metL*	bifunctional aspartate kinase/homoserine dehydrogenase II	2.26	0.0000	/	/
BAU10_05995	*vllY*	4-hydroxyphenylpyruvate dioxygenase	2.24	0.0000	2.39	0.0004
BAU10_05095	*hisA*	phosphoribosylformimino-5-aminoimidazole carboxamide ribotide isomerase	2.24	0.0070	/	/
BAU10_18450	*mmsA*	methylmalonate-semialdehyde dehydrogenase (acylating)	2.18	0.0000	2.50	0.0000
BAU10_00730	*leuC*	3-isopropylmalate dehydratase large subunit	2.16	0.0000	/	/
BAU10_05090	*hisH*	imidazole glycerol phosphate synthase subunit HisH	2.11	0.0000	/	/
BAU10_08970	*trpD*	anthranilate phosphoribosyltransferase	2.10	0.0208	/	/
BAU10_14585	*lysA*	diaminopimelate decarboxylase	2.10	0.0003	/	/
BAU10_00725	*leuD*	3-isopropylmalate dehydratase small subunit	2.08	0.0000	/	/
BAU10_08965	*trpG*	aminodeoxychorismate/anthranilate synthase component II	2.08	0.0003	/	/
BAU10_16330	*speA*	arginine decarboxylase	2.07	0.0000	2.99	0.0000
BAU10_01440	*thrA*	bifunctional aspartate kinase/homoserine dehydrogenase I	1.98	0.0011	/	/
BAU10_16415	*lhgD*	L-2-hydroxyglutarate oxidase	1.97	0.0003	/	/
BAU10_04100	*aroA*	5-enolpyruvylshikimate-3-phosphate synthase	1.96	0.0000	/	/
BAU10_14280	*aroQ*	3-dehydroquinate dehydratase	1.94	0.0000	/	/
BAU10_05535	*dsdA*	D-serine ammonia-lyase	1.94	0.0022	/	/
BAU10_05105	*hisI*	bifunctional phosphoribosyl-AMP cyclohydrolase/phosphoribosyl-ATP diphosphatase	1.92	0.0007	/	/
BAU10_01320	*dapB*	4-hydroxy-tetrahydrodipicolinate reductase	1.91	0.0000	/	/
BAU10_05835	*astE*	succinylglutamate desuccinylase	1.89	0.0000	/	/
BAU10_21925	*Sfri_1503*	NAD(P)-dependent oxidoreductase	1.87	0.0000	/	/

Locus tag	Symbol	Gene product	P-FC	FDR	TR-FC	FDR
BAU10_05665	*hutG*	formimidoylglutamase	1.87	0.0142	2.73	0.0000
BAU10_15060	*pheC*	cyclohexadienyl dehydratase	1.82	0.0000	/	/
BAU10_04925	*ald*	alanine dehydrogenase	1.73	0.0000	7.10	0.0000
BAU10_14075	*cysE*	serine acetyltransferase	1.71	0.0000	/	/
BAU10_18430	*Mccc2*	methylcrotonoyl-CoA carboxylase	1.66	0.0140	/	/
BAU10_18420	*mccA*	acetyl/propionyl/methylcrotonyl-CoA carboxylase subunit alpha	1.65	0.0126	/	/
BAU10_07155	*cobB*	NAD-dependent protein deacylase	1.65	0.0000	/	/
BAU10_08975	*trpC*	bifunctional indole-3-glycerol-phosphate synthase	1.63	0.0005	/	/
BAU10_05075	*hisD*	histidinol dehydrogenase	1.62	0.0000	/	/
BAU10_01445	*thrB*	homoserine kinase	1.62	0.0000	/	/
BAU10_12485	*nadB*	L-aspartate oxidase	1.60	0.0000	/	/
BAU10_07355	*msrC*	GAF domain-containing protein	1.58	0.0006	/	/
BAU10_06525	*ectA*	L-2,4-diaminobutyric acid acetyltransferase	1.56	0.0150	/	/
BAU10_05660	*hutU*	urocanate hydratase	1.55	0.0000	3.08	0.0000
BAU10_18985	*katG2*	catalase/peroxidase HPI	1.54	0.0000	/	/
BAU10_20150	*ltaE*	low-specificity L-threonine aldolase	1.53	0.0000	/	/
BAU10_05080	*hisC*	histidinol-phosphate aminotransferase	1.52	0.0158	/	/
BAU10_05930	*dapA*	dihydrodipicolinate synthase family protein	1.52	0.0000	3.41	0.0000
BAU10_10050	*aroC*	chorismate synthase	1.52	0.0007	/	/
BAU10_14205	*aspA*	aspartate ammonia-lyase	-38.70	0.0000	-19.59	0.0000
BAU10_13665	*argB*	acetylglutamate kinase	-13.26	0.0024	5.10	0.0000
BAU10_13660	*argG*	argininosuccinate synthase	-8.20	0.0001	/	/
BAU10_09145	*metE*	methionine synthase	-4.65	0.0205	-4.17	0.0000
BAU10_17230	*ansB*	L-asparaginase 2	-4.54	0.0000	-5.57	0.0000
BAU10_13670	*argC*	N-acetyl-gamma-glutamyl-phosphate reductase	-4.21	0.0009	17.48	0.0000
BAU10_08615	*sdaA*	L-serine dehydratase 1	-4.16	0.0000	-2.99	0.0000
BAU10_20100	*VPA1001*	alanine racemase	-2.89	0.0002	-2.09	0.0000

Locus tag	Symbol	Gene product	P-FC	FDR	TR-FC	FDR
BAU10_13655	*argH*	argininosuccinate lyase	-2.20	0.0013	8.22	0.0000
BAU10_03070	*cysK*	cysteine synthase A	-2.06	0.0018	/	/
BAU10_08875	*rhbA*	diaminobutyrate--2-oxoglutarate transaminase family protein	-1.83	0.0000	/	/
BAU10_03675	*alaA*	aminotransferase class I/II-fold pyridoxal phosphate-dependent enzyme	/	/	-3.10	0.0000
BAU10_20390	*sdaC*	HAAAP family serine/threonine permease	/	/	2.89	0.0000
BAU10_03190	*asnB*	asparagine synthetase B	/	/	-7.33	0.0000
BAU10_05670	*hutI*	imidazolonepropionase	/	/	2.36	0.0001
BAU10_05655	*hutH*	histidine ammonia-lyase	/	/	2.57	0.0000
BAU10_24270	*cadA*	lysine decarboxylase LdcC	/	/	-3.05	0.0018
BAU10_12815	*davD*	NAD-dependent succinate-semialdehyde dehydrogenase	/	/	-2.01	0.0000
BAU10_06535	*ectC*	ectoine synthase	/	/	2.25	0.0008
BAU10_18980	*gltB*	glutamate synthase	/	/	5.07	0.0000
BAU10_02385	*pepD*	aminoacyl-histidine dipeptidase	/	/	-2.74	0.0000
BAU10_16325	*speB*	agmatinase	/	/	2.18	0.0000
BAU10_13125	*argF*	Ornithine carbamoyltransferase	/	/	14.01	0.0000
BAU10_17355	*OCC_04335*	aminotransferase, classes I and II superfamily	/	/	-6.21	0.0000
BAU10_06140	*davD*	NAD-dependent succinate-semialdehyde dehydrogenase	/	/	1.18	0.0004
**nucleoside metabolism**						
BAU10_23775	*ppnP*	pyrimidine/purine nucleoside phosphorylase	18.70	0.0491	4.47	0.0000
BAU10_14395	*sthA*	pyridine nucleotide transhydrogenase	4.65	0.0000	2.91	0.0000
BAU10_01875	*PA2428*	polyphosphate kinase 2	4.38	0.0000	2.11	0.0000
BAU10_11135	*deoD1*	purine nucleoside phosphorylase	2.22	0.0000	2.16	0.0000
BAU10_23565	*VVA0006*	GTP cyclohydrolase II	2.19	0.0015	/	/
BAU10_16860	--	ADP-ribosylglycohydrolase	2.15	0.0000	/	/
BAU10_13890	--	ExeM/NucH family extracellular endonuclease	1.95	0.0000	2.14	0.0000
BAU10_19750	*pntB*	pyridine nucleotide transhydrogenase	1.73	0.0040	/	/
BAU10_01995	*ndk*	nucleoside diphosphate kinase	1.72	0.0000	/	/

Locus tag	Symbol	Gene product	P-FC	FDR	TR-FC	FDR
BAU10_12415	*relA*	GTP pyrophosphokinase	1.69	0.0000	/	/
BAU10_24250	*purH*	bifunctional phosphoribosylaminoimidazolecarboxamide formyltransferase/IMP cyclohydrolase	1.60	0.0000	/	/
BAU10_02515	*ppnN*	LOG family protein	1.58	0.0391	/	/
BAU10_05365	*nutA*	bifunctional UDP-sugar hydrolase/5'-nucleotidase	1.56	0.0000	/	/
BAU10_09470	*btuR*	cob(I)yrinic acid a,c-diamide adenosyltransferase	1.53	0.0010	/	/
BAU10_16465	*HI_0519*	nucleoside permease NupC	-60.02	0.0087	/	/
BAU10_12620	*dns*	deoxyribonuclease I	-2.37	0.0000	/	/
BAU10_19395	*HI_0206*	bifunctional metallophosphatase/5'-nucleotidase	-2.32	0.0000	/	/
BAU10_01330	*carB*	carbamoyl-phosphate synthase large subunit	-2.07	0.0000	/	/
BAU10_18945	--	endonuclease/exonuclease/phosphatase family protein	-1.75	0.0000	/	/
BAU10_07325	*pyrD*	dihydroorotate dehydrogenase 2	-1.73	0.0000	/	/
BAU10_01725	*VP0554*	inosine/xanthosine triphosphatase	-1.67	0.0009	/	/
BAU10_20145	--	NUDIX hydrolase	-1.63	0.0111	/	/
BAU10_19840	*nrdD*	anaerobic ribonucleoside-triphosphate reductase	-1.50	0.0000	/	/
BAU10_19315	*gsk*	inosine-guanosine kinase	/	/	-2.46	0.0000
BAU10_00990	--	HNH endonuclease	/	/	2.69	0.0000
**lipid metabolism**						
BAU10_11100	*fadD15*	AMP-binding protein	5.03	0.0000	3.77	0.0000
BAU10_20650	*thlA*	ACAT, atoB; acetyl-CoA C-acetyltransferase	4.16	0.0016	3.75	0.0000
BAU10_21815	--	acyl-CoA thioester hydrolase-related protein	3.99	0.0008	/	/
BAU10_20620	*fabG*	3-ketoacyl-(acyl-carrier-protein) reductase	3.86	0.0001	/	/
BAU10_20630	*hibch*	enoyl-CoA hydratase	3.61	0.0000	2.52	0.0003
BAU10_20640	*Acad8*	acyl-CoA dehydrogenase	3.24	0.0163	2.43	0.0000
BAU10_05455	*fadE*	acyl-CoA dehydrogenase	3.00	0.0000	2.11	0.0001
BAU10_18445	*thlA*	ACAT, atoB; acetyl-CoA C-acetyltransferase	2.76	0.0003	/	/
BAU10_18320	*HI_0002*	putative long-chain-fatty-acid-CoA ligase	2.75	0.0001	/	/

Locus tag	Symbol	Gene product	P-FC	FDR	TR-FC	FDR
BAU10_20635	*yngF*	enoyl-CoA hydratase	2.74	0.0000	3.37	0.0000
BAU10_10085	*fadI*	acetyl-CoA C-acyltransferase FadI	2.66	0.0000	/	/
BAU10_09835	*fadH*	NADPH-dependent 2,4-dienoyl-CoA reductase	2.60	0.0007	2.10	0.0002
BAU10_19425	*fadL*	outer membrane protein transport protein	2.50	0.0000	/	/
BAU10_19130	*ZK262.3*	lipase	2.38	0.0043	/	/
BAU10_11005	*glpQ*	glycerophosphoryl diester phosphodiesterase	2.31	0.0000	5.83	0.0000
BAU10_10080	*fadJ*	fatty acid oxidation complex subunit alpha FadJ	2.28	0.0000	/	/
BAU10_19420	*volA*	lipase	2.25	0.0000	/	/
BAU10_15270	*fadB*	fatty acid oxidation complex subunit alpha FadB	2.22	0.0000	3.05	0.0000
BAU10_18760	*aslA*	arylsulfatase	2.12	0.0000	7.96	0.0000
BAU10_18460	*paaF*	enoyl-CoA hydratase	1.97	0.0000	/	/
BAU10_18455	*ACAD8*	acyl-CoA dehydrogenase	1.95	0.0000	3.01	0.0179
BAU10_17095	*clsC*	phospholipase D family protein	1.92	0.0005	/	/
BAU10_10115	*mlaA*	vacJ lipoprotein	1.67	0.0000	/	/
BAU10_15265	*fadA*	acetyl-CoA C-acyltransferase FadA	1.62	0.0000	2.75	0.0000
BAU10_01580	*lgt*	prolipoprotein diacylglyceryl transferase	1.55	0.0012	/	/
BAU10_03405	*fadD*	long-chain-fatty-acid--CoA ligase FadD	1.52	0.0023	/	/
BAU10_03460	*fabF*	beta-ketoacyl synthase, C-terminal domain protein	-1.75	0.0117	/	/
BAU10_19945	*fabH2*	beta-ketoacyl-ACP synthase III	-1.67	0.0000	/	/
BAU10_03465	*fabG*	3-ketoacyl-(acyl-carrier-protein) reductase	-1.62	0.0015	/	/
BAU10_02170	*glpA*	sn-glycerol-3-phosphate dehydrogenase subunit A	/	/	2.20	0.0000
BAU10_09355	*yjjU*	patatin family protein	/	/	2.34	0.0000
BAU10_02165	*glpB*	anaerobic glycerol-3-phosphate dehydrogenase, subunit B	/	/	2.06	0.0004
**protein metabolism**						
BAU10_23935	*NLN*	M3 family oligoendopeptidase	10.24	0.0000	2.81	0.0000
BAU10_06455	*ypwA*	peptidase M32	10.16	0.0000	4.39	0.0000
BAU10_05890	--	peptidase S41	9.86	0.0000	3.94	0.0000

Locus tag	Symbol	Gene product	P-FC	FDR	TR-FC	FDR
BAU10_05830	--	oligoendopeptidase F	8.58	0.0000	3.03	0.0000
BAU10_12025	*glnB*	Nitrogen regulatory protein P-II	8.46	0.0084	/	/
BAU10_21450	*f1pep1*	S9 family peptidase	6.55	0.0000	2.49	0.0000
BAU10_19575	--	peptidase M15	6.37	0.0414	5.27	0.0000
BAU10_13875	*hipO*	amidohydrolase	5.55	0.0006	3.21	0.0000
BAU10_22780	*dacC*	D-alanyl-D-alanine carboxypeptidase	4.41	0.0256	8.78	0.0000
BAU10_03145	--	selenoprotein W-related protein	4.39	0.0000	/	/
BAU10_16315	*ptrB*	S9 family peptidase	4.34	0.0000	2.52	0.0000
BAU10_14730	*ggt*	gamma-glutamyltransferase	4.15	0.0000	3.70	0.0000
BAU10_24055	*slr1322*	peptidase C69	4.04	0.0000	2.94	0.0000
BAU10_18935	--	glutathione S-transfersae-related protein	3.98	0.0046	2.13	0.0001
BAU10_07335	*pepN*	aminopeptidase N	3.71	0.0000	/	/
BAU10_01990	*pepB*	aminopeptidase PepB	3.38	0.0000	2.48	0.0000
BAU10_22310	*f1pep1*	S9 family peptidase	3.13	0.0000	2.07	0.0000
BAU10_13590	--	AAA family ATPase	3.10	0.0002	/	/
BAU10_05960	*APP2*	aminopeptidase P family protein	2.99	0.0000	/	/
BAU10_08765	--	peptidase	2.75	0.0002	/	/
BAU10_15500	*prlC*	oligopeptidase A	2.73	0.0000	2.00	0.0000
BAU10_14775	*ampp*	aminopeptidase P family protein	2.63	0.0000	/	/
BAU10_20320	--	putative glutathione S-transferase	2.36	0.0000	2.86	0.0000
BAU10_08755	--	imelysin family protein	2.05	0.0000	2.72	0.0000
BAU10_22295	*ycbZ*	Putative Lon protease homolog	2.01	0.0000	/	/
BAU10_01105	*glnE*	bifunctional [glutamate--ammonia ligase]-adenylyl-L-tyrosine phosphorylase/[glutamate--ammonia-ligase] adenylyltransferase	1.90	0.0365	/	/
BAU10_01635	*fkpB*	peptidyl-prolyl cis-trans isomerase, FKBP-type	1.78	0.0002	/	/
BAU10_03370	*TRYP7*	serine protease	1.65	0.0060	/	/
BAU10_13085	*pepA*	leucyl aminopeptidase	1.59	0.0000	/	/

Locus tag	Symbol	Gene product	P-FC	FDR	TR-FC	FDR
BAU10_22890	*MGYG_04702*	putative carboxypeptidase G2	-3.64	0.0000	-2.45	0.0000
BAU10_17870	*pepT*	peptidase T	-1.55	0.0300		
BAU10_10915	*csdE*	cysteine desulfurase sulfur acceptor subunit CsdE	/	/	-2.40	0.0164
BAU10_16430	--	cystatin	/	/	5.08	0.0000
BAU10_00285	*hslV*	ATP-dependent protease HslV	/	/	2.05	0.0000
BAU10_23305	--	putative lactoylglutathione lyase	/	/	3.03	0.0002
**other secondary metabolites and compounds**						
BAU10_14375	*btuB*	TonB-dependent vitamin B12 receptor	9.91	0.0000	3.12	0.0000
BAU10_12535	*ygfA*	5-formyltetrahydrofolate cyclo-ligase	5.57	0.0000	/	/
BAU10_20115	*dld*	D-lactate dehydrogenase	5.56	0.0000	2.73	0.0000
BAU10_03375	*purU*	formyltetrahydrofolate deformylase	5.43	0.0000	/	/
BAU10_06045	--	glyoxalase	4.00	0.0092	/	/
BAU10_12585	*epd*	D-erythrose 4-phosphate dehydrogenase	2.76	0.0000	/	/
BAU10_16925	*Bphyt_4144*	nitronate monooxygenase	2.68	0.0078	/	/
BAU10_00230	--	putative 5-carboxymethyl-2-hydroxymuconate delta isomerase	2.62	0.0000	/	/
BAU10_13255	*HI_1556*	D-2-hydroxyacid dehydrogenase	2.15	0.0000	/	/
BAU10_09125	*isoc1*	isochorismatase family protein	2.06	0.0001	/	/
BAU10_01040	*folB*	putative dihydroneopterin aldolase FolB	1.91	0.0074	/	/
BAU10_06570	--	haloacid dehalogenase-like hydrolase	1.88	0.0000	3.11	0.0000
BAU10_06575	*aslA*	arylsulfatase A	1.88	0.0000	/	/
BAU10_01900	*aceA*	isocitrate lyase	1.87	0.0000	2.60	0.0000
BAU10_13690	*metF*	5,10-methylenetetrahydrofolate reductase	1.83	0.0089	/	/
BAU10_09400	*pabC*	aminodeoxychorismate lyase	1.67	0.0003	/	/
BAU10_11030	*glpD*	aerobic glycerol-3-phosphate dehydrogenase	1.65	0.0000	2.99	0.0000
BAU10_16950	*pncA*	isochorismatase family protein	1.61	0.0000	/	/
BAU10_10380	*yffB*	putative arsenate reductase	1.61	0.0000	/	/

Locus tag	Symbol	Gene product	P-FC	FDR	TR-FC	FDR
BAU10_01035	*plsY*	glycerol-3-phosphate 1-O-acyltransferase PlsY	1.59	0.0028	/	/
BAU10_18960	--	putative pyrophosphohydrolase, MutT family protein	1.57	0.0000	/	/
BAU10_07405	*cysG*	ferrochelatase	1.56	0.0000	/	/
BAU10_19875	*PhzF*	phenazine biosynthesis protein PhzF	1.54	0.0089	/	/
BAU10_00485	*cysG*	uroporphyrinogen-III C-methyltransferase	-5.34	0.0010	-2.62	0.0000
BAU10_00505	*cysC*	adenylylsulfate kinase	-4.86	0.0001	/	/
BAU10_00495	*cysN*	sulfate adenylyltransferase subunit CysN	-2.78	0.0000	/	/
BAU10_09250	*tsdA*	c-type cytochrome	-2.37	0.0000	-2.50	0.0000
BAU10_05315	*hcp*	hydroxylamine reductase	-1.66	0.0158	/	/
BAU10_09615	*moaB*	molybdenum cofactor biosynthesis protein B	/	/	-2.94	0.0000
BAU10_09610	*moaC*	molybdenum cofactor biosynthesis protein C	/	/	-3.12	0.0000
BAU10_24020	*thiE*	thiamine-phosphate diphosphorylase	/	/	-5.13	0.0004
BAU10_09620	*moaA*	GTP 3',8-cyclase MoaA	/	/	-2.50	0.0000
BAU10_09600	*moaE*	molybdenum cofactor biosynthesis protein E	/	/	-2.15	0.0000
BAU10_24130	*coaA*	pantothenate kinase	/	/	-2.37	0.0000
**Transport**						
**carbon transport**						
BAU10_15965	*btuB*	TonB-dependent receptor	12.77	0.0000	5.42	0.0000
BAU10_16760	*mltD*	lytic transglycosylase domain-containing protein	7.09	0.0013	/	/
BAU10_11015	*glpF*	glycerol uptake facilitator protein GlpF	4.33	0.0000	5.04	0.0000
BAU10_06945	*kpsD*	sugar ABC transporter substrate-binding protein	2.74	0.0031	3.02	0.0000
BAU10_01570	*ptsP*	phosphoenolpyruvate-protein phosphotransferase	2.10	0.0000	/	/
BAU10_00855	*mtlA*	PTS mannitol transporter subunit IICBA	2.01	0.0004	/	/
BAU10_11010	*glpT*	glycerol-3-phosphate transporter	1.75	0.0014	4.34	0.0000
BAU10_05800	*tcyP*	sodium/dicarboxylate symporter	-3.80	0.0002	-2.56	0.0000
BAU10_18850	*dcuC*	C4-dicarboxylate transporter, anaerobic	-1.89	0.0022	/	/
BAU10_02930	--	Fe3+-citrate ABC transporter substrate-binding protein	/	/	2.73	0.0093

Locus tag	Symbol	Gene product	P-FC	FDR	TR-FC	FDR
BAU10_14210	*dcuA*	anaerobic C4-dicarboxylate transporter	/	/	-2.47	0.0000
BAU10_22320	*HI_1218*	putative L-lactate permease	/	/	2.91	0.0000
BAU10_19230	*fruA*	PTS fructose transporter subunit IIBC	/	/	4.70	0.0000
**amino acid transport**						
BAU10_20545	*proX*	glycine betaine/proline transport system substrate-binding protein	6.02	0.0002	5.64	0.0000
BAU10_03995	*aotP*	arginine/ornithine transport system ATP-binding protein	5.55	0.0000	2.90	0.0000
BAU10_18315	*braF*	branched-chain amino acid transport system ATP-binding protein	3.50	0.0010	/	/
BAU10_23395	*putP*	sodium/proline symporter PutP	3.38	0.0206	/	/
BAU10_04000	*argT*	amino acid ABC transporter, periplasmic amino acid-binding protein	3.07	0.0000	8.56	0.0000
BAU10_07565	*artI*	arginine transport system substrate-binding protein	2.64	0.0022	6.27	0.0000
BAU10_00875	*metQ*	D-methionine transport system substrate-binding protein	2.59	0.0036	/	/
BAU10_07425	*aapP*	general L-amino acid transport system ATP-binding protein	2.30	0.0000	2.92	0.0000
BAU10_18340	*braG*	branched-chain amino acid transport system ATP-binding protein	2.22	0.0452	2.71	0.0009
BAU10_07410	*aapJ*	general L-amino acid transport system substrate-binding protein	2.18	0.0000	9.80	0.0000
BAU10_18500	*proX*	glycine betaine/proline transport system substrate-binding protein	2.06	0.0059	/	/
BAU10_02545	*metQ*	D-methionine transport system substrate-binding protein	1.92	0.0001	/	/
BAU10_18335	*livK*	branched-chain amino acid transport system substrate-binding protein	1.87	0.0000	2.81	0.0000
BAU10_06495	*ousX*	L-proline glycine betaine binding ABC transporter protein ProX	1.83	0.0000	2.49	0.0000
BAU10_06505	*ousV*	ABC superfamily (glycine/betaine/proline transport protein)	1.81	0.0000	2.41	0.0000
BAU10_23570	--	putative amino acid ABC transporter, periplasmic amino acid-binding portion	1.55	0.0000	4.53	0.0000
BAU10_20535	*artP*	arginine transport system ATP-binding protein	-6.24	0.0013	39.96	0.0000
BAU10_15175	*patH*	amino acid ABC transporter, periplasmic amino acid-binding portion	-5.56	0.0000	/	/
BAU10_20530	*artI*	arginine/ornithine transport system substrate-binding protein	-3.30	0.0038	4.48	0.0000
BAU10_15165	*tcyC*	amino acid ABC transporter ATP-binding protein	-2.37	0.0000	/	/
BAU10_07415	*yhdX*	amino acid ABC transporter, permease protein	/	/	3.13	0.0000
BAU10_17010	*opuAA*	glycine betaine/L-proline ABC transporter ATP-binding protein	/	/	2.11	0.0000
BAU10_09005	*dctA*	proton/glutamate symporter	/	/	2.19	0.0001

Locus tag	Symbol	Gene product	P-FC	FDR	TR-FC	FDR
**peptide transport**						
BAU10_09575	*oppF*	oligopeptide ABC transporter, ATP-binding protein	21.28	0.0000	7.00	0.0000
BAU10_09580	*oppD*	oligopeptide transporter ATP-binding component	19.03	0.0000	5.95	0.0000
BAU10_09595	*oppA*	oligopeptide ABC transporter substrate-binding protein OppA	15.23	0.0000	5.29	0.0000
BAU10_09585	*oppC*	oligopeptide ABC transporter, permease protein	10.90	0.0001	5.91	0.0000
BAU10_15400	*dppC*	peptide ABC transporter, permease protein	8.77	0.0000	7.50	0.0000
BAU10_15385	*gsiA*	peptide ABC transporter, ATP-binding protein	8.74	0.0000	5.63	0.0000
BAU10_22305	*yejF*	oligopeptide transport system ATP-binding protein	5.87	0.0002	/	/
BAU10_15395	*dppB*	peptide ABC transporter, permease protein	5.86	0.0436	6.91	0.0000
BAU10_05965	*oppF*	oligopeptide ABC transporter, ATP-binding protein	5.24	0.0415	/	/
BAU10_15390	*mppA*	ABC transporter substrate-binding protein	4.44	0.0000	8.36	0.0000
BAU10_05975	*oppC*	ABC transporter permease subunit	3.77	0.0054	/	/
BAU10_23470	*oppA*	oligopeptide ABC transporter substrate-binding protein OppA	3.16	0.0000	/	/
BAU10_05970	*oppD*	ATP-binding cassette domain-containing protein	2.59	0.0019	/	/
BAU10_05985	*oppA*	peptide ABC transporter substrate-binding protein	2.17	0.0000	/	/
BAU10_21580	*dppA*	ABC transporter substrate-binding protein	1.83	0.0000	/	/
BAU10_05335	*ynjD*	ABC transporter ATP-binding protein	1.80	0.0404	/	/
BAU10_03180	*yclF*	peptide MFS transporter	-1.93	0.0304	/	/
BAU10_09590	*oppB*	oligopeptide permease ABC transporter membrane protein	/	/	6.48	0.0000
BAU10_15975	*yejE*	putative peptide ABC transporter, permease protein	/	/	3.34	0.0000
BAU10_17105	*nolG*	efflux RND transporter permease subunit	/	/	2.36	0.0000
**ion transport**						
BAU10_09770	*mleN*	Na+/H+ antiporter	-132.74	0.0157	-15.39	0.0000
BAU10_23175	*fiu*	TonB-dependent siderophore receptor	6.62	0.0000	2.20	0.0000
BAU10_18690	*hgbA*	TonB-dependent siderophore receptor	6.60	0.0000	/	/
BAU10_06745	*fct*	TonB-dependent siderophore receptor	6.49	0.0008	/	/
BAU10_13795	*kefB*	glutathione-regulated potassium-efflux system protein KefB	4.96	0.0058	/	/

Locus tag	Symbol	Gene product	P-FC	FDR	TR-FC	FDR
BAU10_23170	*fecA*	TonB-dependent siderophore vibrioferrin receptor PvuA	4.82	0.0000	/	/
BAU10_14235	*H16_A2524*	sodium/solute symporter	3.57	0.0142	/	/
BAU10_22870	*PH1295*	sodium:dicarboxylate symporter	3.38	0.0004	2.06	0.0000
BAU10_13800	*kefG*	glutathione-regulated potassium-efflux system ancillary protein KefG	2.63	0.0016	/	/
BAU10_22005	*ktrA*	potassium transporter TrkA	2.30	0.0000	/	/
BAU10_23745	*nhaD*	Sodium:proton antiporter NhaD	1.93	0.0204	/	/
BAU10_03195	*ybaL*	glutathione-regulated potassium-efflux system protein KefB, putative	1.92	0.0000	/	/
BAU10_07225	*fixI*	cadmium-translocating P-type ATPase	1.86	0.0000	2.28	0.0000
BAU10_13715	*bfr*	bacterioferritin	1.76	0.0000	7.23	0.0000
BAU10_21785	*fatB*	siderophore ABC transporter substrate-binding protein	1.60	0.0001	/	/
BAU10_19540	*hgbA*	TonB-dependent hemoglobin/transferrin/lactoferrin family receptor	1.59	0.0010	/	/
BAU10_13450	*nhaP*	sodium:proton antiporter	1.53	0.0287	/	/
BAU10_04730	*citP*	Na(+)citrate OH(-) antiporter	-7.13	0.0001	-4.71	0.0000
BAU10_00500	*SAC1*	putative sodium/sulfate symporter	-5.81	0.0180	/	/
BAU10_07860	--	cation transporter	-5.68	0.0003	/	/
BAU10_03330	*feoA*	ferrous iron transport protein A	-3.17	0.0128	/	/
BAU10_02340	*abgT*	AbgT family transporter	-2.85	0.0310	/	/
BAU10_01485	*yaaJ*	sodium/alanine symporter	-2.63	0.0009	/	/
BAU10_22030	*fhuC*	ABC transporter ATP-binding protein	-1.66	0.0278	/	/
BAU10_09605	*moaD*	molybdopterin synthase sulfur carrier subunit	-1.51	0.0007	-2.04	0.0130
BAU10_03825	*zntA*	zinc/cadmium/mercury/lead-transporting ATPase	/	/	-2.97	0.0000
BAU10_03135	--	CBS domain-containing protein	/	/	2.71	0.0000
BAU10_02900	*copA*	copper-translocating P-type ATPase	/	/	-2.14	0.0000
BAU10_23655	*gltS*	sodium/glutamate symporter	/	/	-2.01	0.0000
BAU10_12015	*futA1*	Iron-utilization periplasmic protein	/	/	3.65	0.0000
BAU10_23855	*viuB*	putative siderophore utilization protein	/	/	2.46	0.0000
**transport,antibiotic resistance**						

Locus tag	Symbol	Gene product	P-FC	FDR	TR-FC	FDR
BAU10_17090	--	putative tellurite resistance protein-related protein	26.48	0.0155	8.30	0.0000
BAU10_11920	*nolG*	putative multidrug resistance protein	17.96	0.0000	4.01	0.0000
BAU10_23360	*HI_1051*	ATP-binding cassette, subfamily B, multidrug efflux pump	7.42	0.0001	2.78	0.0000
BAU10_09175	*macB*	Macrolide export ATP-binding/permease protein MacB	4.75	0.0023	-2.43	0.0000
BAU10_09170	*macB*	Macrolide export ATP-binding/permease protein MacB	2.73	0.0098	/	/
BAU10_10750	--	tellurite resistance TerB family protein	2.56	0.0026	3.41	0.0000
BAU10_09185	*macA*	efflux RND transporter periplasmic adaptor subunit	2.54	0.0000	-2.92	0.0000
BAU10_09165	*macB*	macrolide ABC transporter ATP-binding protein	2.25	0.0275	/	/
BAU10_03755	*HI_0895*	multidrug efflux RND transporter permease subunit VmeF	1.62	0.0002	/	/
BAU10_17135	--	acriflavin resistance protein	1.55	0.0243	/	/
BAU10_09910	--	antibiotic biosynthesis monooxygenase	-3.13	0.0000	-2.71	0.0000
BAU10_22020	*HI_1051*	ATP-binding cassette, subfamily B, multidrug efflux pump	-1.64	0.0240	/	/
BAU10_22700	--	fusaric acid resistance family protein			4.34	0.0000
**other transport systems**						
BAU10_17100	--	efflux RND transporter periplasmic adaptor subunit	40.59	0.0032	5.56	0.0000
BAU10_15970	*yejB*	microcin C transport system permease protein	33.96	0.0065	3.29	0.0000
BAU10_09280	*oppA*	microcin C transport system substrate-binding protein	18.37	0.0000	12.10	0.0000
BAU10_15955	--	biopolymer transporter ExbB	14.91	0.0000	5.90	0.0000
BAU10_15980	*DR_1571*	microcin C transport system substrate-binding protein	14.39	0.0000	2.91	0.0000
BAU10_11925	--	efflux transporter periplasmic adaptor subunit	6.90	0.0011	3.32	0.0000
BAU10_16405	--	TAXI family TRAP transporter solute-binding subunit	6.46	0.0000	5.38	0.0000
BAU10_00625	*bcsP31*	TAXI family TRAP transporter solute-binding subunit	5.58	0.0000	5.19	0.0000
BAU10_00620	--	TRAP transporter permease	5.20	0.0017	2.22	0.0000
BAU10_10730	*abgT*	efflux pump component MtrF	4.33	0.0001	/	/
BAU10_01360	*btuF*	vitamin B12 ABC transporter substrate-binding protein BtuF	4.07	0.0000	/	/
BAU10_19505	*ybbP*	FtsX-like permease family protein	3.27	0.0455	/	/
BAU10_09545	--	ABC transporter substrate-binding protein	2.63	0.0011	3.41	0.0000

Locus tag	Symbol	Gene product	P-FC	FDR	TR-FC	FDR
BAU10_09535	*ecfA2*	Energy-coupling factor transporter ATP-binding protein	2.50	0.0009	/	/
BAU10_19500	*ybbA*	ABC transporter ATP-binding protein	2.47	0.0000	/	/
BAU10_05345	*ynjB*	ABC transporter solute-binding protein	2.32	0.0229	/	/
BAU10_06180	--	sulfate ABC transporter permease	2.23	0.0395	4.55	0.0000
BAU10_05265	*nolG*	putative efflux system protein	1.85	0.0000	/	/
BAU10_21665	*hutX*	heme utilization cystosolic carrier protein HutX	1.78	0.0000	/	/
BAU10_05255	--	efflux RND transporter periplasmic adaptor subunit	1.76	0.0000	/	/
BAU10_23450	*apxIB*	toxin secretion ATP-binding protein	1.71	0.0000	/	/
BAU10_20840	*modF*	molybdate ABC transporter ATP-binding protein ModF	1.67	0.0364	/	/
BAU10_09710	*VP2115*	TRAP transporter large permease subunit	1.57	0.0102	/	/
BAU10_05920	--	spermidine/putrescine ABC transporter substrate-binding protein	1.50	0.0241	/	/
BAU10_18840	--	OFA family MFS transporter	-3.98	0.0195	/	/
BAU10_22235	*VPA1482*	ABC transporter ATP-binding protein	-3.98	0.0015	/	/
BAU10_10765	*yhdH*	Putative sodium-dependent transporter.	-3.57	0.0326	/	/
BAU10_22240	*EcfT*	energy-coupling factor transporter transmembrane protein EcfT	-1.94	0.0226	/	/
BAU10_18025	*iutA*	TonB-dependent receptor	-1.55	0.0083	/	/
BAU10_06760	*mdlA*	ATP-binding cassette domain-containing protein	/	/	26.02	0.0000
BAU10_00995	--	AAA family ATPase	/	/	4.25	0.0000
BAU10_10155	*ccmB*	heme exporter protein B	/	/	-2.00	0.0002
BAU10_20010	*fdhC*	formate transporter	/	/	2.55	0.0000
BAU10_15350	*CT0075*	Cytochrome c5	/	/	2.83	0.0000
BAU10_17830	*napC*	periplasmic nitrate reductase, cytochrome c-type protein	/	/	3.12	0.0000
BAU10_21060	*abcE*	ATP-binding cassette domain-containing protein	/	/	2.34	0.0000
BAU10_08600	--	putative guanylate cyclase-related protein	/	/	4.01	0.0000
BAU10_06755	*mdlB*	ATP-binding cassette domain-containing protein	/	/	4.53	0.0000
BAU10_01475	--	cation-binding protein	/	/	3.37	0.0000
BAU10_21960	*BB3856*	azurin	/	/	2.04	0.0000

Locus tag	Symbol	Gene product	P-FC	FDR	TR-FC	FDR
BAU10_07165	*potD*	ABC transporter, periplasmic spermidine putrescine-binding protein PotD	/	/	2.05	0.0000
**bacterial secretion system**						
BAU10_20270	*ytxE*	type VI secretion system protein TssL	10.66	0.0012	5.87	0.0000
BAU10_07865	*CesT*	type III secretion chaperone CesT	6.42	0.0349	/	/
BAU10_20945	*HopJ*	HopJ type III effector protein	5.84	0.0005	2.44	0.0014
BAU10_20285	--	type VI secretion system-associated FHA domain protein TagH	5.48	0.0000	3.76	0.0000
BAU10_20265	*tssM1*	type VI secretion system membrane subunit TssM	3.91	0.0000	4.79	0.0000
BAU10_20280	*VasD*	type VI secretion lipoprotein/VasD	3.39	0.0005	5.87	0.0000
BAU10_20200	*vgrG1b*	type VI secretion system tip protein VgrG	3.27	0.0057	4.43	0.0000
BAU10_20260	*tagF*	type VI secretion system-associated protein TagF	2.93	0.0022	3.96	0.0000
BAU10_20275	--	type VI secretion system-associated protein	2.78	0.0000	4.98	0.0000
BAU10_20240	*tssC2*	type VI secretion system contractile sheath large subunit	2.61	0.0000	3.56	0.0000
BAU10_20245	*tssB1*	type VI secretion protein	2.57	0.0018	4.08	0.0000
BAU10_20210	*clpV1*	type VI secretion system ATPase TssH	2.38	0.0000	3.42	0.0000
BAU10_22715	*aaeA*	HlyD family secretion protein	2.08	0.0001	3.89	0.0000
BAU10_23440	*lapP*	T1SS associated transglutaminase-like cysteine proteinase LapP	1.96	0.0007	/	/
BAU10_20205	*hcp*	type VI secretion system secreted protein Hcp	1.73	0.0242	4.53	0.0000
BAU10_13380	*exeD*	MSHA biogenesis protein MshL	1.72	0.0010	/	/
BAU10_13360	*tapC*	type II secretion system F family protein	1.54	0.0000	/	/
BAU10_13375	*exeA*	MSHA biogenesis protein MshM	1.50	0.0116	/	/
BAU10_07795	--	putative type III secretion protein	-172362.71	0.0017	/	/
BAU10_07750	*yopD*	type III secretion system translocon subunit VopD	-10.76	0.0027	/	/
BAU10_07930	*sctC*	type III secretion system outer membrane ring subunit SctC	-5.54	0.0050	/	/
BAU10_07940	--	T3SS regulon anti-activator ExsD family protein	-5.33	0.0122	-2.01	0.0005
BAU10_17785	*secD*	protein translocase subunit SecD	-2.46	0.0000	/	/
BAU10_20235	*tssC1*	type VI secretion system contractile sheath large subunit	/	/	3.51	0.0000

Locus tag	Symbol	Gene product	P-FC	FDR	TR-FC	FDR
BAU10_20250	--	type VI secretion system protein TssA	/	/	4.12	0.0000
BAU10_20220	*tssF1*	VAT2t	/	/	3.15	0.0000
BAU10_17845	--	NapD protein	/	/	4.83	0.0000
BAU10_19415	--	YgdI/YgdR family lipoprotein	/	/	6.19	0.0021
**outermembrane and other membrane proteins**						
BAU10_15940	*tonB*	energy transducer TonB	40.02	0.0004	4.30	0.0000
BAU10_05495	*lysO*	membrane protein	10.22	0.0008	/	/
BAU10_04045	--	porin, putative	6.17	0.0000	2.40	0.0000
BAU10_14175	--	putative periplasmic protein CpxP	5.31	0.0343	4.48	0.0000
BAU10_23740	--	putative inner membrane protein	4.05	0.0000	3.27	0.0000
BAU10_17000	*ompV*	MipA/OmpV family protein	3.93	0.0000	/	/
BAU10_21200	--	outer membrane protein N, non-specific porin	3.89	0.0000	2.20	0.0000
BAU10_23890	*ompW*	outer membrane protein OmpW	3.56	0.0000	8.04	0.0000
BAU10_14975	--	putative periplasmic protein	3.46	0.0009	/	/
BAU10_16310	--	putative outer membrane protein	3.35	0.0000	8.92	0.0000
BAU10_08670	--	porin family protein	2.93	0.0000	2.07	0.0000
BAU10_11095	--	Outer membrane receptor for Fe3+-dicitrate	2.46	0.0339	3.82	0.0000
BAU10_06865	--	porin family protein	2.42	0.0000	/	/
BAU10_03100	*qmcA*	SPFH/Band 7/PHB domain protein	2.35	0.0000	/	/
BAU10_07480	*pal*	putative outer membrane protein	2.04	0.0000	3.47	0.0000
BAU10_08035	*jhp_1381*	predicted membrane fusion protein	1.98	0.0000	/	/
BAU10_17505	--	inner membrane protein YccF	1.94	0.0095	/	/
BAU10_17905	*ompA*	outer membrane protein A	1.91	0.0000	13.67	0.0000
BAU10_21370	*ybhS*	putative transmembrane protein	1.88	0.0452	/	/
BAU10_07490	--	agglutination protein	1.81	0.0000	/	/
BAU10_22195	*ybgA*	membrane protein	1.72	0.0000	/	/
BAU10_05625	*oprF*	Outer membrane protein	1.70	0.0005	/	/

Locus tag	Symbol	Gene product	P-FC	FDR	TR-FC	FDR
BAU10_05260	*mdtA*	putative periplasmic linker protein	1.65	0.0000	/	/
BAU10_22175	--	outer membrane lipoprotein	1.65	0.0025	/	/
BAU10_06950	--	OmpA family protein	1.64	0.0350	2.36	0.0031
BAU10_04270	*tolR*	TolR membrane protein	1.63	0.0342	/	/
BAU10_01715	*slt*	murein transglycosylase	1.57	0.0000	/	/
BAU10_11035	*zntB*	putative membrane transport protein	1.53	0.0026	/	/
BAU10_12590	*irgA*	ligand-gated channel protein	-14.86	0.0001	/	/
BAU10_02925	*ompA*	outer membrane protein OmpA	-4.33	0.0000	-2.32	0.0000
BAU10_22895	*yjiG*	membrane protein	-4.13	0.0111	-2.53	0.0000
BAU10_22900	*yjiH*	membrane protein	-3.64	0.0000	-2.64	0.0000
BAU10_02890	--	porin	-2.55	0.0000	/	/
BAU10_10105	*ompP1*	outer membrane protein transport protein	-2.00	0.0005	-2.71	0.0000
BAU10_19055	--	sel1 repeat family protein	/	/	2.35	0.0000
BAU10_22025	*foxA*	TonB-dependent siderophore receptor	/	/	2.24	0.0000
BAU10_16690	--	porin family protein	/	/	-2.98	0.0000
BAU10_09360	--	outer membrane beta-barrel protein	/	/	2.78	0.0000
BAU10_05390	--	membrane protein	/	/	2.49	0.0000
BAU10_02905	*chiP*	putative chitoporin	/	/	2.37	0.0000
BAU10_06550	--	tetratricopeptide repeat protein	/	/	3.87	0.0000
BAU10_23950	*HI_1015*	GntP family permease	/	/	4.33	0.0000
BAU10_22435	--	PepSY domain-containing protein	/	/	2.90	0.0000
BAU10_07595	*artI*	binding protein component ofABC transporter	/	/	7.21	0.0000
BAU10_21260	*BpOF4_10220*	YjiH family protein	/	/	2.05	0.0000
**Replication,recombination and repair**						
BAU10_19590	*pcrA*	uvrD, pcrA; DNA helicase II / ATP-dependent DNA helicase PcrA	5.51	0.0003	4.31	0.0000
BAU10_07435	*parB*	chromosome partitioning protein ParB	37.35	0.0000	9.26	0.0000

Locus tag	Symbol	Gene product	P-FC	FDR	TR-FC	FDR
BAU10_01430	*RraA*	ribonuclease E inhibitor RraA	9.93	0.0000	4.98	0.0000
BAU10_19630	--	DEAD/DEAH box helicase	6.74	0.0000	4.04	0.0000
BAU10_19635	*rep*	DNA helicase	4.78	0.0000	3.93	0.0000
BAU10_19625	*MJ1519*	ecD; exodeoxyribonuclease V alpha subunit	4.07	0.0000	3.62	0.0000
BAU10_00115	*NGR_a03120*	IS66 family transposase	4.02	0.0156	4.23	0.0000
BAU10_17860	--	RNA helicase	3.25	0.0190	/	/
BAU10_19615	*yqhH*	DEAD/DEAH box helicase family protein	3.08	0.0000	2.92	0.0000
BAU10_19605	*yqhH*	DEAD/DEAH box helicase	2.51	0.0000	2.63	0.0000
BAU10_19700	--	site-specific integrase	2.00	0.0417	2.17	0.0000
BAU10_06305	*ywqA*	DEAD/DEAH box helicase	1.84	0.0009	/	/
BAU10_17120	--	DNAase	1.63	0.0000	2.49	0.0000
BAU10_04365	*samB*	translesion error-prone DNA polymerase V subunit UmuC	-7.79	0.0382	/	/
BAU10_11555	*umuC*	DNA polymerase V subunit UmuC	-7.79	0.0382	/	/
BAU10_15620	*rmuC*	DNA recombination protein RmuC	-2.10	0.0000	/	/
BAU10_15425	--	chromosome partitioning protein ParA	-2.08	0.0031	/	/
BAU10_08905	*uvrC*	excinuclease ABC subunit UvrC	-1.70	0.0002	/	/
BAU10_14890	*yrdD*	DNA topoisomerase	-1.59	0.0097	/	/
BAU10_09935	*recR*	recombination protein RecR	/	/	2.08	0.0000
BAU10_19680	*yrrC*	AAA family ATPase	/	/	2.94	0.0000
**Transcription**						
BAU10_04065	*cspD*	cold shock-like protein CspD	165.34	0.0059	9.36	0.0000
BAU10_02405	*crl*	transcriptional regulator Crl	6.91	0.0000	3.76	0.0000
BAU10_05620	*DnaJ*	molecular chaperone	6.50	0.0086	/	/
BAU10_19715	*dmlR*	LysR family transcriptional regulator	5.52	0.0000	/	/
BAU10_11070	*csgD*	response regulator transcription factor	5.38	0.0000	/	/
BAU10_20510	*rbcR*	LysR family transcriptional regulator	5.34	0.0126		

Locus tag	Symbol	Gene product	P-FC	FDR	TR-FC	FDR
BAU10_04930	*lrp*	Leucine-responsive regulatory protein, regulator for leucine (or lrp) regulon and high-affinity branched-chain amino acid transport system	5.30	0.0000	2.09	0.0000
BAU10_22065	*csgD*	helix-turn-helix transcriptional regulator	5.24	0.0000	4.37	0.0000
BAU10_08130	*ybaA*	RNA signal recognition particle	3.77	0.0000	/	/
BAU10_13400	*csrD*	RNase E specificity factor CsrD	3.46	0.0000	/	/
BAU10_12360	*rpoS*	DNA-directed RNA polymerase, sigma subunit	3.20	0.0038	2.01	0.0002
BAU10_19195	--	transcriptional regulator, HTH_3 family	3.09	0.0062	/	/
BAU10_20415	--	transcriptional regulator	3.02	0.0006	/	/
BAU10_20585	--	transcriptional regulator, LysR family protein	2.52	0.0022	/	/
BAU10_00295	*cytR*	DNA-binding transcriptional regulator CytR	2.30	0.0004	/	/
BAU10_18370	*budR*	LysR family transcriptional regulator	2.17	0.0002	/	/
BAU10_05900	*pdhR*	putative transcription regulator	2.11	0.0000	/	/
BAU10_24200	*rsd*	regulator of sigma D	2.10	0.0003	/	/
BAU10_17285	*ydcR*	GntR family transcriptional regulator	1.94	0.0184	2.03	0.0001
BAU10_21500	--	PAS factor	1.94	0.0000	/	/
BAU10_08860	--	glycine cleavage system regulatory protein	1.88	0.0000	2.28	0.0000
BAU10_14360	*RBM34*	RNA-binding protein	1.79	0.0000	2.41	0.0000
BAU10_18485	*betI*	transcriptional regulator, TetR family protein	1.72	0.0149	/	/
BAU10_23370	*lrp*	transcriptional regulator, AsnC family protein	1.70	0.0019	/	/
BAU10_21705	--	LuxT	1.61	0.0000	/	/
BAU10_10875	*rpoE*	putative RNA polymerase ECF-type sigma factor	1.54	0.0101	/	/
BAU10_06615	*glcR*	DeoR/GlpR transcriptional regulator	1.54	0.0000	/	/
BAU10_05235	*pspF*	psp operon transcriptional activator	1.53	0.0000	/	/
BAU10_05725	--	putative transcriptional regulator	1.53	0.0000	/	/
BAU10_20120	*HI_1364*	LysR family transcriptional regulator	1.51	0.0138	/	/
BAU10_05250	*pspC*	phage shock protein C	1.51	0.0002	/	/
BAU10_07760	*lcrH*	low calcium response locus protein H	-7.38	0.0002	/	/

Locus tag	Symbol	Gene product	P-FC	FDR	TR-FC	FDR
BAU10_13685	--	predicted transcriptional regulator	-2.00	0.0004	/	/
BAU10_17945	--	sugar-binding transcriptional regulator	-1.88	0.0000	/	/
BAU10_15505	*asnC*	transcriptional regulator AsnC	-1.85	0.0001	/	/
BAU10_17735	*slyA*	MarR family transcriptional regulator	-1.76	0.0179	/	/
BAU10_23905	--	Rho-specific inhibitor of transcription termination (YaeO)	/	/	3.00	0.0028
BAU10_05760	*ihfA*	integration host factor alpha subunit	/	/	2.17	0.0000
BAU10_21305	*ptxR*	LysR family transcriptional regulator	/	/	-2.07	0.0007
BAU10_19895	*oxyR*	LysR family transcriptional regulator	/	/	-5.18	0.0000
BAU10_01605	*hlyU*	transcriptional activator HlyU	/	/	-2.09	0.0000
BAU10_21230	*aaeR*	transcriptional regulator	/	/	-2.05	0.0000
BAU10_15210	*ibpA*	16 kDa heat shock protein A	/	/	2.97	0.0000
BAU10_01600	*nhaR*	transcriptional activator protein NhaR	/	/	-2.88	0.0000
BAU10_06690	--	transcriptional regulator, LysR family protein	/	/	-2.60	0.0000
BAU10_16390	*dctD*	sigma-54-dependent Fis family transcriptional regulator	/	/	2.59	0.0000
BAU10_05450	--	transcriptional regulator, TetR family	/	/	2.24	0.0002
BAU10_20850	--	transcriptional regulator	/	/	-2.02	0.0001
BAU10_07945	*exsA*	transcriptional regulator ExsA	/	/	-2.04	0.0328
BAU10_22220	*yiaW*	DUF3302 domain-containing protein	/	/	2.16	0.0000
**translation**						
BAU10_12265	*oadA1*	sodium-extruding oxaloacetate decarboxylase subunit alpha	8.95	0.0000	/	/
BAU10_22785	*pip*	alpha/beta fold hydrolase	4.14	0.0000	/	/
BAU10_03780	--	D-Tyr-tRNAtyr deacylase	2.53	0.0000	/	/
BAU10_08560	*rimJ*	ribosomal-protein-alanine acetyltransferase	2.41	0.0001	/	/
BAU10_07295	*rmf*	ribosome modulation factor	1.92	0.0157	/	/
BAU10_16345	*rimK*	ribosomal protein S6 modification protein	1.83	0.0003	3.70	0.0000
BAU10_00355	*rpmC*	50S ribosomal protein L29	-3.43	0.0000	/	/
BAU10_00690	*rsmA*	dimethyladenosine transferase	-1.69	0.0026	/	/

Locus tag	Symbol	Gene product	P-FC	FDR	TR-FC	FDR
BAU10_16340	*rimK*	ribosomal protein S6 modification protein	/	/	2.91	0.0000
BAU10_17045	*fusA*	elongation factor G	/	/	2.05	0.0002
BAU10_05375	*rplY*	50S ribosomal protein L25	/	/	2.55	0.0000
BAU10_09440	*yceD*	23S rRNA accumulation protein YceD	/	/	2.00	0.0000
BAU10_02305	*dnaK*	molecular chaperone DnaK	/	/	2.04	0.0000
BAU10_14155	*groS1*	10 kDa chaperonin protein GroES	/	/	2.01	0.0005
BAU10_20255	*prpC*	probable phosphoprotein phosphatase	/	/	3.61	0.0000
BAU10_03105	*cnoX*	co-chaperone YbbN	/	/	2.59	0.0000
**post-translational modification**						
BAU10_23990	--	GNAT family N-acetyltransferase	9.15	0.0343	2.41	0.0041
BAU10_02115	*yhcX*	GNAT family N-acetyltransferase	8.86	0.0000	/	/
BAU10_22670	*ysnE*	putative acetyltransferase	7.39	0.0003	4.05	0.0000
BAU10_15760	*yiiD*	GNAT family N-acetyltransferase	6.28	0.0000	/	/
BAU10_18965	--	GNAT family N-acetyltransferase	6.23	0.0058	/	/
BAU10_22675	--	GNAT family N-acetyltransferase	5.90	0.0000	3.14	0.0000
BAU10_22265	*aaaT*	putative acetyltransferase	2.47	0.0009	2.30	0.0000
BAU10_20970	--	class I SAM-dependent methyltransferase	1.77	0.0000	/	/
BAU10_01400	*yhcC*	TIGR01212 family radical SAM protein	-3.01	0.0026	/	/
BAU10_05370	--	GNAT family N-acetyltransferase	/	/	2.72	0.0001
BAU10_24000	*yoaA*	acetyltransferase	/	/	2.51	0.0000
BAU10_04920	--	methyltransferase	/	/	-2.05	0.0000
**<u>celluar process</u>**						
**cell growth and death**						
BAU10_18525	*AF_1420*	putative stomatin-like protein	3.01	0.0031	/	/
BAU10_19765	--	late competence development ComFB family protein	2.08	0.0000	3.31	0.0000
BAU10_22805	*sll1483*	secreted and surface protein containing fasciclin-like repeat	1.84	0.0154	2.37	0.0041
BAU10_01165	*zapE*	cell division protein ZapE	1.80	0.0000	2.14	0.0000

Locus tag	Symbol	Gene product	P-FC	FDR	TR-FC	FDR
BAU10_13310	*mreC*	rod shape-determining protein MreC	1.55	0.0000	/	/
BAU10_18800	--	VWA domain-containing protein	/	/	2.95	0.0000
**cell motility**						
**Flagellar assembly**						
BAU10_10240	*fliP*	flagellar type III secretion system pore protein FliP	1.56	0.0449	/	/
BAU10_03990	--	54K polar flagellar sheath protein A	1.52	0.0000	/	/
BAU10_10345	*flaB*	flagellin	-1.54	0.0021	/	/
BAU10_22540	*flhA*	flagellar biosynthesis protein	/	/	-2.78	0.0000
**Bacterial chemotaxis**						
BAU10_12230	*luxS*	Autoinducer-2 production protein LuxS	7.30	0.0000	3.75	0.0000
BAU10_19335	*tcpI*	methyl-accepting chemotaxis protein	4.86	0.0000	2.18	0.0000
BAU10_21265	*pctA*	methyl-accepting chemotaxis protein	2.75	0.0000	/	/
BAU10_23635	*PA2652*	chemotaxis protein	2.39	0.0091	/	/
BAU10_06660	*cheY*	chemotaxis protein CheY	2.18	0.0000	2.60	0.0000
BAU10_22725	*ydiK*	AI-2E family transporter	1.96	0.0398	3.46	0.0000
BAU10_03840	*ctpH*	methyl-accepting chemotaxis protein	1.71	0.0004	/	/
BAU10_16035	*PA2652*	methyl-accepting chemotaxis protein	1.51	0.0007	/	/
BAU10_20095	*ctpH*	methyl-accepting chemotaxis protein	-105.07	0.0002	-23.27	0.0000
BAU10_22290	*nahY*	methyl-accepting chemotaxis protein	-4.05	0.0000	/	/
BAU10_09875	*bdlA*	methyl-accepting chemotaxis protein	-2.08	0.0007	/	/
BAU10_17925	*aer*	methyl-accepting chemotaxis protein	-1.54	0.0000	/	/
BAU10_04855	*PA2652*	chemotaxis protein	-1.53	0.0260	/	/
BAU10_22080	*pctC*	methyl-accepting chemotaxis protein	/	/	-2.01	0.0000
BAU10_21020	*bdlA*	methyl-accepting chemotaxis protein	/	/	2.64	0.0006
**cell response to stress and stimulus**						
BAU10_20450	*RP573*	copper resistance protein CopZ	4.07	0.0000	4.03	0.0000
BAU10_19030	--	universal stress protein	3.63	0.0000	2.55	0.0000

Locus tag	Symbol	Gene product	P-FC	FDR	TR-FC	FDR
BAU10_21100	--	glutaredoxin	2.62	0.0004	/	/
BAU10_23290	*ahpC*	alkyl hydroperoxide reductase c22 protein	2.17	0.0000	/	/
BAU10_23280	*ACIAD3023*	organic hydroperoxide resistance protein	2.12	0.0003	/	/
BAU10_18240	*uspE*	universal stress protein	1.96	0.0000	/	/
BAU10_12255	*CEQORH*	quinone oxidoreductase	1.89	0.0000	/	/
BAU10_21315	--	redoxin family protein	1.87	0.0000	/	/
BAU10_21920	*katA*	catalase	1.87	0.0000	/	/
BAU10_22395	*sodC*	superoxide dismutase	1.79	0.0015	2.45	0.0000
BAU10_00705	*lptD*	organic solvent tolerance protein precursor	1.74	0.0017	/	/
BAU10_14190	*sodA*	superoxide dismutase, Mn	1.67	0.0000	/	/
BAU10_15530	*uspA*	universal stress protein A	1.56	0.0005	/	/
BAU10_19725	*uspA*	universal stress protein	/	/	4.47	0.0000
BAU10_19805	--	NirD/YgiW/YdeI family stress tolerance protein	/	/	13.06	0.0000
BAU10_01660	*HI_1126.1*	putative carbon starvation protein A	/	/	2.06	0.0000
BAU10_21460	*uspA*	universal stress protein A	/	/	2.15	0.0000
BAU10_19890	*ydeP*	FdhF/YdeP family oxidoreductase	/	/	-4.68	0.0000
**signal transduction**						
BAU10_20290	*prkC*	serine/threonine protein kinase	9.25	0.0000	5.38	0.0000
BAU10_03610	*dctD*	C4-dicarboxylate transport transcriptional regulatory protein	4.86	0.0049	2.93	0.0000
BAU10_20650	*thlA*	ACAT, atoB; acetyl-CoA C-acetyltransferase	4.16	0.0016	3.75	0.0000
BAU10_15680	*cc4*	cytochrome c4	3.88	0.0157	3.87	0.0000
BAU10_14220	*agmR*	putative two-component response regulator	3.78	0.0000	3.65	0.0000
BAU10_08015	*citT*	response regulator	3.70	0.0000	2.05	0.0002
BAU10_10655	*glnD*	[protein-PII] uridylyltransferase	3.48	0.0000	/	/
BAU10_01185	*petB*	ubiquinol-cytochrome c reductase, cytochrome b	3.22	0.0020	3.36	0.0000
BAU10_01180	*petA*	ubiquinol-cytochrome c reductase, iron-sulfur subunit	3.12	0.0001	2.63	0.0000
BAU10_08595	*LuxQ*	Autoinducer 2 sensor kinase/phosphatase LuxQ	3.01	0.0140	2.20	0.0000

Locus tag	Symbol	Gene product	P-FC	FDR	TR-FC	FDR
BAU10_01190	*petC*	ubiquinol-cytochrome c reductase, cytochrome c1	2.95	0.0035	4.18	0.0000
BAU10_18445	*thlA*	ACAT, atoB; acetyl-CoA C-acetyltransferase	2.76	0.0003	/	/
BAU10_07250	*ccoN1*	cytochrome c oxidase, subunit CcoN	2.72	0.0221	3.34	0.0000
BAU10_15715	*glnG*	nitrogen regulation protein	2.66	0.0000	/	/
BAU10_11615	*PA1727*	bifunctional diguanylate cyclase/phosphodiesterase	2.64	0.0063	/	/
BAU10_10550	*glnB*	nitrogen regulatory protein P-II	2.57	0.0011	/	/
BAU10_07245	*CcoO*	cytochrome c oxidase, subunit CcoO	2.48	0.0000	3.98	0.0000
BAU10_07235	*ccoP2*	cytochrome c oxidase, subunit CcoP	2.46	0.0001	3.83	0.0000
BAU10_19775	*cph2*	putative regulatory component of sensory transduction system	2.42	0.0061	/	/
BAU10_23575	*dhkK*	hybrid sensor histidine kinase/response regulator	2.40	0.0000	2.83	0.0000
BAU10_07240	*CcoQ*	cytochrome c oxidase, subunit CcoQ	2.40	0.0000	4.67	0.0000
BAU10_06190	*dhkJ*	hybrid sensor histidine kinase/response regulator	2.35	0.0000	9.11	0.0000
BAU10_09465	*slr0328*	protein-tyrosine phosphatase	2.29	0.0000	/	/
BAU10_09780	--	histidine phosphotransferase	2.27	0.0000	/	/
BAU10_21030	--	tandem-95 repeat protein	1.95	0.0000	3.71	0.0000
BAU10_15730	*glnA*	glutamine synthetase	1.90	0.0000	/	/
BAU10_18870	*cqsS*	hybrid sensor histidine kinase/response regulator	1.84	0.0000	/	/
BAU10_23445	--	diguanylate cyclase	1.77	0.0000	/	/
BAU10_08760	--	c-type cytochrome	1.73	0.0000	2.42	0.0000
BAU10_11050	--	histidine kinase	1.71	0.0005	/	/
BAU10_10100	--	chemotaxis protein	1.62	0.0005	/	/
BAU10_22835	*gfcE*	wza, gfcE; polysaccharide biosynthesis/export protein	1.61	0.0000	/	/
BAU10_18300	*etfa*	electron transfer flavoprotein subunit alpha	1.60	0.0007	4.41	0.0000
BAU10_22845	*wzc*	putative tyrosine kinase	1.57	0.0000	/	/
BAU10_14185	*cpxA*	two-component sensor protein	1.54	0.0027	/	/
BAU10_06930	*luxO*	sigma-54-dependent Fis family transcriptional regulator	1.53	0.0412	7.27	0.0000
BAU10_01835	*phoR*	histidine protein kinase PhoR	1.53	0.0111	/	/

Locus tag	Symbol	Gene product	P-FC	FDR	TR-FC	FDR
BAU10_04740	*citD*	citrate lyase acyl carrier protein	-6.92	0.0001	-4.15	0.0000
BAU10_04750	*citF*	citrate lyase subunit alpha	-6.47	0.0000	-4.35	0.0000
BAU10_04745	*citE*	citrate (pro-3S)-lyase subunit beta	-5.97	0.0000	-4.23	0.0000
BAU10_04735	*citC*	[citrate [pro-3S]-lyase] ligase	-5.18	0.0000	-2.44	0.0000
BAU10_04755	*citX*	holo-ACP synthase CitX	-4.64	0.0323	-2.86	0.0000
BAU10_17725	*luxP*	autoinducer 2-binding periplasmic protein LuxP	-2.29	0.0000	/	/
BAU10_09230	*ttrB*	tetrathionate reductase subunit B	-1.86	0.0182	-2.22	0.0000
BAU10_04240	*cydA*	cytochrome D ubiquinol oxidase, subunit I	-1.65	0.0145	/	/
BAU10_09235	*ttrC*	tetrathionate reductase subunit C	-1.64	0.0008	-2.16	0.0000
BAU10_01420	*arcA*	aerobic respiration control protein FexA	-1.63	0.0088	/	/
BAU10_09240	*ttrA*	tetrathionate reductase subunit A	-1.55	0.0000	/	/
BAU10_09960	*rssB*	response regulator	/	/	2.06	0.0000
BAU10_03155	*toxS*	ToxS	/	/	-2.00	0.0000
BAU10_10800	*pleD*	response regulator	/	/	2.20	0.0000
BAU10_17695	*NIK1*	response regulator	/	/	2.03	0.0001
BAU10_14255	*pleC*	hybrid sensor histidine kinase/response regulator	/	/	2.43	0.0000
BAU10_20835	*nahY*	aerotaxis sensor receptor protein	/	/	3.44	0.0000
BAU10_10065	*sixA*	phosphohistidine phosphatase	/	/	2.00	0.0000
BAU10_14525	*cdgJ*	EAL domain-containing protein	/	/	-2.11	0.0000
BAU10_21050	*tolC*	TolC family protein	/	/	2.96	0.0000
BAU10_22545	--	diguanylate phosphodiesterase	/	/	-2.61	0.0000
BAU10_04760	*citG*	2-(5''-triphosphoribosyl)-3'-dephospho-CoA synthase	/	/	-3.43	0.0000
BAU10_01985	*iscX*	Fe-S cluster assembly protein IscX	/	/	2.31	0.0001
BAU10_20050	*yvbT*	luciferase	/	/	3.81	0.0000
**virulence**						
BAU10_05555	--	agglutination protein	7.66	0.0000	4.44	0.0000
BAU10_19975	*iutA*	ferric aerobactin receptor precursor	4.77	0.0000	/	/

Locus tag	Symbol	Gene product	P-FC	FDR	TR-FC	FDR
BAU10_10495	--	Attachment to host cells and virulence	3.03	0.0111	/	/
BAU10_12225	*yfjD*	putative hemolysin	2.75	0.0000	/	/
BAU10_07475	--	agglutination protein	2.19	0.0026	3.09	0.0000
BAU10_12090	*lpxL*	LpxL/LpxP family Kdo(2)-lipid IV(A) lauroyl/palmitoleoyl acyltransferasee	1.93	0.0089	-2.22	0.0000
BAU10_07145	--	immunity 49 family protein	1.91	0.0000	2.08	0.0000
BAU10_06585	--	RTX toxin	/	/	3.17	0.0000
**Unknown function and hypothetical protein**						
BAU10_15960	--	DUF3450 domain-containing protein	40.88	0.0000	10.87	0.0000
BAU10_16975	--	methyltransferase domain-containing protein	25.89	0.0341	13.97	0.0000
BAU10_23730	--	hypothetical protein	24.02	0.0028	2.39	0.0000
BAU10_01480	--	DUF3545 family protein	18.97	0.0379	4.15	0.0015
BAU10_19620	--	hypothetical protein	8.44	0.0001	3.66	0.0000
BAU10_19720	--	hypothetical protein	7.88	0.0013	2.08	0.0000
BAU10_04305	*yjfL*	DUF350 domain-containing protein	7.64	0.0231	/	/
BAU10_18695	--	DUF2218 domain-containing protein	7.49	0.0000	/	/
BAU10_15935	--	TPR domain protein	6.32	0.0451	3.16	0.0000
BAU10_16890	--	glyoxalase	5.92	0.0056	3.23	0.0000
BAU10_19585	--	DUF1998 domain-containing protein	5.26	0.0000	3.57	0.0000
BAU10_19580	--	hypothetical protein	5.05	0.0000	3.56	0.0000
BAU10_19595	--	hypothetical protein	5.04	0.0000	4.44	0.0000
BAU10_19675	--	ATP-binding protein	4.85	0.0000	4.33	0.0000
BAU10_00745	--	SH3 domain-containing protein	4.82	0.0082	2.18	0.0000
BAU10_19405	--	hypothetical protein	4.65	0.0000	/	/
BAU10_19600	--	AAA family ATPase	4.58	0.0000	4.09	0.0000
BAU10_24060	*slr0863*	TldD/PmbA family protein	4.45	0.0002	2.49	0.0000
BAU10_17040	--	hypothetical protein	4.38	0.0003	8.77	0.0000
BAU10_17685	--	hypothetical protein	4.30	0.0007	4.22	0.0000

Locus tag	Symbol	Gene product	P-FC	FDR	TR-FC	FDR
BAU10_13050	--	hypothetical protein	4.09	0.0282	/	/
BAU10_16550	--	hypothetical protein	3.81	0.0008	/	/
BAU10_09270	--	hypothetical protein	3.80	0.0010	3.39	0.0000
BAU10_09725	--	hypothetical protein	3.65	0.0172	/	/
BAU10_23345	*yiiM*	MOSC domain-containing protein	3.63	0.0000	/	/
BAU10_14705	--	gene 3 protein-related protein	3.62	0.0000	/	/
BAU10_07740	*bcsP31*	31 kDa immunogenic protein	3.39	0.0000	1.47	0.0000
BAU10_20300	--	hypothetical protein	3.26	0.0165	2.79	0.0000
BAU10_20230	--	protein of avirulence locus	3.24	0.0000	1.52	0.0000
BAU10_09180	--	TolC family protein	3.22	0.0056	-3.46	0.0000
BAU10_15710	*PA1727*	EAL domain-containing protein	3.21	0.0000	/	/
BAU10_05885	--	hypothetical protein	3.01	0.0001	3.61	0.0000
BAU10_14125	--	hypothetical protein	3.00	0.0000	/	/
BAU10_19610	*MJECS02*	hypothetical protein	2.89	0.0000	3.02	0.0000
BAU10_09065		EAL domain-containing protein	2.85	0.0000	2.24	0.0005
BAU10_09815	--	hypothetical protein	2.85	0.0000	4.55	0.0000
BAU10_07140	*tse5*	RHS repeat protein	2.85	0.0086	2.01	0.0000
BAU10_00965	--	DUF4935 domain-containing protein	2.84	0.0000	/	/
BAU10_22705	--	TolC family protein	2.83	0.0330	4.94	0.0000
BAU10_05715	--	helix-turn-helix domain-containing protein	2.79	0.0162	/	/
BAU10_10375	--	conserved hypothetical protein	2.79	0.0002	/	/
BAU10_11445	--	hypothetical protein	2.78	0.0000	/	/
BAU10_03095	--	NfeD family protein	2.70	0.0131	/	/
BAU10_06490	*HI_1246*	LTA synthase family protein	2.62	0.0002	/	/
BAU10_03130	--	putative nitrogen regulatory protein P-II family protein	2.59	0.0025	4.51	0.0000
BAU10_07710	--	GFA family protein	2.57	0.0001	3.40	0.0000
BAU10_12235	--	hypothetical protein	2.50	0.0136	/	/

Locus tag	Symbol	Gene product	P-FC	FDR	TR-FC	FDR
BAU10_00685	*apaG*	Protein apaG	2.48	0.0000	/	/
BAU10_01665	--	DUF2799 domain-containing protein	2.39	0.0025	2.08	0.0000
BAU10_05710	*VP1283*	heavy metal-binding domain-containing protein	2.39	0.0437	2.16	0.0000
BAU10_06625	*Ping_1243*	heme-degrading domain-containing protein	2.34	0.0000	/	/
BAU10_01375	--	DUF1499 domain-containing protein	2.32	0.0003	/	/
BAU10_03255	--	DUF1853 family protein	2.28	0.0000	/	/
BAU10_14955	*fixG*	FixG-related protein	2.27	0.0000	2.34	0.0000
BAU10_12675	--	DUF4426 domain-containing protein	2.26	0.0495	2.67	0.0000
BAU10_08200	--	DUF4263 domain-containing protein	2.25	0.0000	/	/
BAU10_19135	--	hypothetical protein	2.22	0.0365	/	/
BAU10_10445	--	DUF2069 domain-containing protein	2.21	0.0274	/	/
BAU10_06215	--	hypothetical protein	2.15	0.0383	3.01	0.0000
BAU10_15575		hypothetical protein	2.15	0.0000	/	/
BAU10_11440	--	hypothetical protein	2.12	0.0029	/	/
BAU10_08115	*HI_1456*	DUF2846 domain-containing protein	2.10	0.0052	/	/
BAU10_09035	*yciI*	YciI family protein	2.10	0.0000	/	/
BAU10_22015	*kdpD*	DUF4118 domain-containing protein	2.06	0.0000	/	/
BAU10_19370	*VPA0850*	YceI family protein	2.03	0.0000	2.27	0.0000
BAU10_12935	--	hypothetical protein	2.03	0.0307	/	/
BAU10_18780	*yeaC*	MoxR family ATPase	1.99	0.0000	2.72	0.0000
BAU10_06185	--	hypothetical protein	1.99	0.0410	11.28	0.0000
BAU10_17115	--	hypothetical protein	1.96	0.0000	2.83	0.0000
BAU10_05490	--	hypothetical protein	1.96	0.0000	2.30	0.0000
BAU10_16970	--	glycine zipper 2TM domain-containing protein	1.95	0.0022	/	/
BAU10_13835	--	DUF1338 domain-containing protein	1.93	0.0000	14.42	0.0000
BAU10_06935	--	Hpt domain-containing protein	1.91	0.0023	4.08	0.0264
BAU10_13275	*ylaK*	PhoH family protein	1.89	0.0499	/	/

Locus tag	Symbol	Gene product	P-FC	FDR	TR-FC	FDR
BAU10_08235	--	hypothetical protein	1.87	0.0097	/	/
BAU10_11690	--	hypothetical protein	1.87	0.0097	/	/
BAU10_20295	--	Hpt domain-containing protein	1.87	0.0025	6.35	0.0000
BAU10_23225	--	DUF302 domain-containing protein, partial	1.87	0.0002	/	/
BAU10_10945	--	LysM peptidoglycan-binding domain-containing protein	1.86	0.0001	/	/
BAU10_00865	--	DUF541 domain-containing protein	1.83	0.0000	/	/
BAU10_07485	--	tandem-95 repeat protein	1.81	0.0000	/	/
BAU10_06985	*VP1481*	phosphotransferase	1.75	0.0002	2.93	0.0000
BAU10_08570	*VP1870*	TIGR01620 family protein	1.74	0.0027	/	/
BAU10_23690	--	DUF3081 family protein	1.73	0.0007	2.41	0.0157
BAU10_07300	--	DUF3466 family protein	1.73	0.0000	/	/
BAU10_21750	*ycbX*	MOSC domain-containing protein	1.72	0.0014	/	/
BAU10_04420	--	ATP-binding protein	1.72	0.0070	/	/
BAU10_08555	--	Predicted hydrolase	1.72	0.0005	/	/
BAU10_23000	--	putative CymC protein	1.71	0.0320	/	/
BAU10_02525	--	hypothetical protein	1.71	0.0025	/	/
BAU10_22270	--	phosphotransferase	1.70	0.0347	/	/
BAU10_06095	--	DUF4336 domain-containing protein	1.70	0.0022	/	/
BAU10_03950	*VP0986*	YeaH/YhbH family protein	1.69	0.0000	9.69	0.0000
BAU10_00210	--	hypothetical protein	1.66	0.0000	/	/
BAU10_17080	--	tetratricopeptide repeat protein	1.66	0.0000	/	/
BAU10_03365	--	hypothetical protein	1.65	0.0136	/	/
BAU10_23125	--	hypothetical protein	1.64	0.0002	/	/
BAU10_03945	*ycgB*	SpoVR family protein	1.64	0.0000	7.95	0.0000
BAU10_20500	*HSP31*	type 1 glutamine amidotransferase domain-containing protein	1.64	0.0000	/	/
BAU10_00140	--	hypothetical protein	1.62	0.0000	/	/
BAU10_16455	--	DUF2913 family protein	1.61	0.0076	/	/

Locus tag	Symbol	Gene product	P-FC	FDR	TR-FC	FDR
BAU10_02375	--	succinylglutamate desuccinylase/aspartoacylase family protein	1.59	0.0000	/	/
BAU10_04535	--	hypothetical protein	1.58	0.0001	/	/
BAU10_11325	--	hypothetical protein	1.58	0.0001	/	/
BAU10_22215	*vdcA*	GGDEF domain-containing protein	1.57	0.0064	/	/
BAU10_23515	--	DUF342 domain-containing protein	1.56	0.0003	/	/
BAU10_11755	--	hypothetical protein	1.53	0.0006	/	/
BAU10_16425	--	hypothetical protein	1.53	0.0242	/	/
BAU10_03955	*yeaG*	conserved hypothetical protein	1.51	0.0000	14.16	0.0000
BAU10_06630	*pleD*	GGDEF domain protein	1.50	0.0006	/	/
BAU10_07800	*tyeA*	TyeA	-19.70	0.0161	/	/
BAU10_07765	*lcrV*	VcrV	-17.79	0.0006	/	/
BAU10_07600	--	DUF3316 domain-containing protein	-11.69	0.0053	/	/
BAU10_04700	--	hypothetical protein VH1709_contig00050-0127	-11.14	0.0056	-4.37	0.0000
BAU10_04765	--	hypothetical protein	-6.65	0.0000	-3.06	0.0000
BAU10_03445	*HI_0519*	NupC family protein	-5.60	0.0007	/	/
BAU10_00945	--	hypothetical protein	-2.95	0.0025	/	/
BAU10_17380	--	cupin	-2.47	0.0271	2.29	0.0005
BAU10_06250	--	hypothetical protein	-2.43	0.0058	/	/
BAU10_15410	--	acid phosphatase	-2.39	0.0055	-2.16	0.0000
BAU10_11365	--	Ynd	-2.24	0.0002	/	/
BAU10_05045	--	hypothetical protein	-2.20	0.0281	/	/
BAU10_02895	--	copper amine oxidase	-1.92	0.0000	/	/
BAU10_17710	--	hypothetical protein	-1.86	0.0018	/	/
BAU10_09900	--	aryl-sulfate sulfotransferase	-1.82	0.0136	2.57	0.0035
BAU10_13775	*slyX*	conserved domain protein	-1.79	0.0157	/	/
BAU10_10740	*trmO*	conserved hypothetical protein	-1.78	0.0034	-2.02	0.0000
BAU10_00535	*ytfJ*	YtfJ protein precursor	-1.77	0.0128	/	/

Locus tag	Symbol	Gene product	P-FC	FDR	TR-FC	FDR
BAU10_08725	--	hypothetical protein	-1.65	0.0407	/	/
BAU10_09275	--	hypothetical protein	-1.63	0.0000	/	/
BAU10_19780	--	DUF3069 domain-containing protein	-1.60	0.0000	/	/
BAU10_17020	*VPA0321*	DUF134 domain-containing protein	-1.59	0.0175	/	/
BAU10_10670	*truC*	YqcC family protein	-1.58	0.0321	/	/
BAU10_02850	*ybaK*	YbaK family protein	-1.51	0.0000	/	/
BAU10_18585	--	hypothetical protein	-1.50	0.0018	/	/
BAU10_06265	*sll1178*	hypothetical protein	/	/	2.16	0.0000
BAU10_19050	--	hypothetical protein	/	/	2.59	0.0000
BAU10_10675	--	DUF3549 family protein	/	/	2.19	0.0000
BAU10_06795	--	hypothetical protein	/	/	6140.00	0.0001
BAU10_19685	--	hypothetical protein	/	/	2.03	0.0366
BAU10_14480	--	DUF2492 family protein	/	/	2.40	0.0000
BAU10_17375	--	DUF465 domain-containing protein	/	/	2.05	0.0011
BAU10_07985	--	hypothetical protein	/	/	2.43	0.0000
BAU10_06260	--	hypothetical protein BAU10_06260	/	/	2.29	0.0000
BAU10_19690	--	hypothetical protein	/	/	2.14	0.0022
BAU10_20460	--	hypothetical protein	/	/	3.47	0.0274
BAU10_17865	--	hypothetical protein	/	/	2.65	0.0012
BAU10_10795	--	DUF2789 domain-containing protein	/	/	2.67	0.0000
BAU10_09850	*yeaC*	DUF1315 family protein	/	/	2.18	0.0001
BAU10_19695	--	hypothetical protein	/	/	2.21	0.0000
BAU10_06480	--	hypothetical protein	/	/	2.10	0.0001
BAU10_16485	--	DUF3612 domain-containing protein	/	/	2.06	0.0081
BAU10_10995	--	GGGtGRT protein	/	/	-2.77	0.0022
BAU10_07230	--	putative analog of CcoH	/	/	2.61	0.0000
BAU10_13070	--	RDD family protein	/	/	2.14	0.0015

Locus tag	Symbol	Gene product	P-FC	FDR	TR-FC	FDR
BAU10_12030	*VP2494*	YacL family protein	/	/	1320.00	0.0348
BAU10_20910	*VF_0530*	DNA-binding protein VF530	/	/	2.18	0.0000
BAU10_16695	*HI_0461*	putative virK protein	/	/	-3.66	0.0000
